# Robust Binding of Disulfide-Substituted Rhenium Bipyridyl Complexes for CO_2_ Reduction on Gold Electrodes

**DOI:** 10.3389/fchem.2020.00086

**Published:** 2020-02-13

**Authors:** Mauricio Cattaneo, Facheng Guo, H. Ray Kelly, Pablo E. Videla, Laura Kiefer, Sara Gebre, Aimin Ge, Qiliang Liu, Shaoxiong Wu, Tianquan Lian, Víctor S. Batista

**Affiliations:** ^1^INQUINOA-UNT-CONICET, Facultad de Bioquímica, Química y Farmacia, Instituto de Química Física, Universidad Nacional de Tucumán, San Miguel de Tucumán, Argentina; ^2^Department of Chemistry, Yale University, New Haven, CT, United States; ^3^Department of Chemistry, Emory University, Atlanta, GA, United States

**Keywords:** disulfide, rhenium complexes, CO_2_ reduction, spectroelectrochemistry, modified gold surfaces, SFG spectroscopy

## Abstract

Heterogenization of homogenous catalysts on electrode surfaces provides a valuable approach for characterization of catalytic processes *in operando* conditions using surface selective spectroelectrochemistry methods. Ligand design plays a central role in the attachment mode and the resulting functionality of the heterogenized catalyst as determined by the orientation of the catalyst relative to the surface and the nature of specific interactions that modulate the redox properties under the heterogeneous electrode conditions. Here, we introduce new [Re(L)(CO)_3_Cl] catalysts for CO_2_ reduction with sulfur-based anchoring groups on a bipyridyl ligand, where L = 3,3′-disulfide-2,2′-bipyridine (SSbpy) and 3,3′-thio-2,2′-bipyridine (Sbpy). Spectroscopic and electrochemical analysis complemented by computational modeling at the density functional theory level identify the complex [Re(SSbpy)(CO)_3_Cl] as a multi-electron acceptor that combines the redox properties of both the rhenium tricarbonyl core and the disulfide functional group on the bipyridyl ligand. The first reduction at −0.85 V (vs. SCE) involves a two-electron process that breaks the disulfide bond, activating it for surface attachment. The heterogenized complex exhibits robust anchoring on gold surfaces, as probed by vibrational sum-frequency generation (SFG) spectroscopy. The binding configuration is normal to the surface, exposing the active site to the CO_2_ substrate in solution. The attachment mode is thus particularly suitable for electrocatalytic CO_2_ reduction.

## Introduction

Electrochemical CO_2_ reduction powered by renewable energy sources, such as wind or solar light, is an attractive pathway for generation of fuels or chemical feedstock (Qiao et al., 2016). Rhenium complexes are effective homogeneous catalysts with high turnover frequencies for CO_2_ to CO conversion (Clark et al., 2018a). However, they typically require high overpotentials. Therefore, there is significant interest in the development of a fundamental understanding of structure/property relations of rhenium polypyridine complexes that can be exploited for the rational design and improvement of catalysts that could operate at lower overpotentials (Schneider et al., 2016). Non-innocent ligands can modulate the redox properties of transition metal complexes, and gives access to several redox states in combination with the metal center (Kaim, 2011). In particular, 2,2′-bipyridine (bpy) ligands have been extensively investigated in coordination chemistry. Here, we explore the functionalization of bpy ligands with disulfide groups in order to develop rhenium complexes for CO_2_ reduction that strongly bind to gold electrode surfaces.

The electrocatalytic reduction of CO_2_ by Re(I) bipyridyl complexes to form CO is highly selective and involves a first reduction of the bpy ligand, followed by a second reduction localized at the rhenium center that activates the catalyst for CO_2_ binding upon loss of the axial halide ligand (Clark et al., 2018a). Heterogenization on electrode surfaces could allow for fundamental studies of the reaction mechanism using surface-selective spectroelectrochemistry methods, as well as the development of catalytic surfaces for selective electrocatalytic CO_2_ reduction. Moreover, structural and electronic effects of the catalyst under working conditions can lend insight into the design and optimization of catalytic materials. An outstanding challenge is the design of robust anchoring ligands that could stabilize the catalysts on the surface even under sufficiently negative potentials as necessary for catalysis. Functional groups on the bpy ligand can be used to anchor the complexes to surfaces, including thiol groups for gold electrodes and ethynyl groups for carbon electrodes (Clark et al., 2018b; Zhanaidarova et al., 2019). The orientation and properties of molecular catalysts on electrode surfaces can be analyzed by combining vibrational sum-frequency generation (SFG) spectroscopy and computational modeling (Anfuso et al., 2011, 2012; Clark et al., 2016, 2018b; Ge et al., 2016, 2017). The resulting insights should be particularly informative for the rational design of catalytic materials (Ge et al., 2019).

Recently, the molecule 3,3′-disulfide-2,2′-bipyridine (SSbpy), with a functional disulfide bridge, was synthesized for the first time showing interesting redox capabilities (Cattaneo et al., 2018). The redox active dithiol/disulfide switch coupled to proton transfer provides the ligand with promising properties for heterogenization as well as multi-electron storage and redox control. Moreover, the sulfur atoms are particularly suited for immobilizing the ligand on gold surfaces. Such a non-innocent ligand in conjunction with the redox state transitions of the metal center results in a two-electron reduction of the complex at a lower potential, enabling the accumulation of redox equivalents at a lower overpotential when compared to complexes with other bpy ligands (Hua et al., submitted). Therefore, the SSbpy ligand in rhenium tricarbonyl complexes allows the complex to accumulate an extra reducing equivalent at the disulfide bond to facilitate stronger binding to gold surfaces than previously reported.

**Scheme 1:**
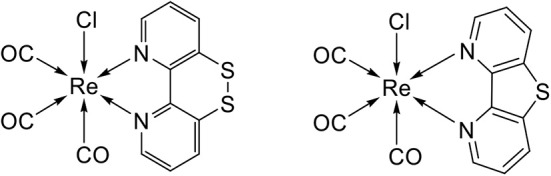
Structure of novel complexes **ReSS** and **ReS** studied in this work.

In this work, we report the synthesis, spectroscopy and electrochemical characterization of [Re(SS-bpy)(CO)_3_Cl] (**ReSS**), a new rhenium catalyst based on the SSbpy non-innocent ligand (Scheme 1). A comparison to the related monosulfur catalyst [Re(S-bpy)(CO)_3_Cl] (**ReS**), that lacks the disulfide bond, highlights the advantages of the dithiol/disulfide switch. Sum-frequency generation spectroscopy and computational modeling allow us to understand the molecular characteristics of these new catalysts as well as their orientation and properties when immobilized on gold electrodes.

## Materials and Methods

### Materials

[Re(CO)_5_Cl] was used as received from Aldrich. The ligands 3,3′-(S)_2_-2,2′-bpy (SSbpy) and 3,3′-S-2,2′-bpy (Sbpy) were obtained as previously reported (Cattaneo et al., 2018). All chemicals used were reagent grade. MeCN was freshly distilled over P_4_O_10_ for electrochemical measurements. Tetrakis(n-butyl)ammonium hexafluorophosphate (TBAH) was dried at 150°C for 24 h before being used as supporting electrolyte in electrochemical measurements.

### Measurements

UV–Vis spectra were recorded on a Varian Cary 50 spectrophotometer, using 1 cm quartz cells. IR spectra were obtained as KBr pellets and in acetonitrile (MeCN) solutions in a liquid cell with CaF_2_ windows using a Perkin–Elmer Spectrum RX-I FTIR spectrometer. Emission measurements were carried out in 1 cm fluorescence cells with a Shimadzu RF-5301 PC spectrofluorometer. The optical density of the solutions at the excitation wavelength was below 0.1 to minimize both inner filtering and re-absorption effects. The fluorescence quantum yields (ϕ_F_) in Ar saturated solutions were determined using [Ru(bpy)_3_]^2+^ in CH_3_CN as an actinometer (ϕ_F_ = 0.095) (Ishida et al., 2010, 2016), by comparing the integrated fluorescence intensities of the samples and reference solutions.

Cyclic voltammetry (CV) and differential pulse voltammetry (DPV) experiments were carried out using BAS Epsilon EC equipment with vitreous C as the working electrode, Pt wire as the auxiliary electrode, and a Ag/AgCl (3 M NaCl) reference electrode. The cyclic voltammograms were measured at a scan rate of 100 mV/s. The solutions used in voltammetry were degassed with Ar prior to each measurement. The IR optically transparent thin layer electrochemical (OTTLE) cell was equipped with CaF_2_ windows, with a Pt-minigrid as the working electrode, a Pt-minigrid as the auxiliary electrode and a Ag wire pseudo-reference electrode (Hartl et al., 1994). Controlled potential electrolysis within the OTTLE cell were carried out using a Teq-04 potentiostat. Spectroelectrochemical measurements in the UV–Vis region were performed by using a Honeycomb spectroelectrochemical cell kit with a Pt or Au-microstructured working electrode from PINE Research Instrumentation, and a Ag/AgCl (saturated KCl) reference electrode. Controlled potential electrolysis with this cell was carried out using the BAS potentiostat. Chemical analyses were performed at INQUIMAE, University of Buenos Aires, Buenos Aires, Argentina, with an estimated error of ±0.5%.

NMR samples for both [Re(SSbpy)(CO)_3_Cl] (6.37 mM) and [Re(Sbpy)(CO)_3_Cl] (10.9 mM) complexes were prepared by rotary evaporating the complex in MeCN, and redissolving in 0.75 mL CD_3_CN obtained from Sigma Aldrich (≥99.8 atom % D). All ^1^H, ^13^C, correlated spectroscopy (COSY), ^1^H-^13^C heteronuclear single quantum coherence spectroscopy (HSQC), ^1^H-^13^C heteronuclear multiple bond correlation (HMBC), and ^1^H-^13^C were acquired from a Bruker Ascend 600 MHz spectrometer with a prodigy cryoprobe (liquid nitrogen cooled). The frequencies for the ^1^H and ^13^C NMR were 600.18 and 150.915 MHz, respectively.

The instrument used for mass spectrometry was a Thermo linear quadrupole ion trap-Fourier transform mass spectrometer (LTQ-FTMS) with a nanospray source and static nanospray probe equipped with a New Objective econo picotip using nitrogen as the backing pressure source. From each sample (1 mM [Re(SSbpy)(CO)_3_Cl] and [Re(Sbpy)(CO)_3_Cl]), 10 μL was loaded onto the picotip, which was then placed into the nanosource where a voltage of 1.0–2.5 kV was applied. The resolution used was 100,000 and the data was analyzed with the Xcalibur program.

### SFG Measurements

A commercial high-repetition-rate femtosecond laser (CARBIDE, Light Conversion, Ltd.) provided 229 fs pulses at 1030 nm with 200 μJ per pulse. Ninety percent of the output was first compressed and then entered into a high power optical parametric amplifier (Orpheus-HP, Light Conversion, Ltd.) to generate a broadband mid-infrared pulse from 1,350 to 16,000 nm. The remaining 10% of the uncompressed 1,030 nm output was directly introduced into the second harmonic band compressor (SHBC, Light Conversion, Ltd.), in which two inversely chirped pulses with the same magnitude were generated from the broadband 1,030 nm pulse and used to produce a narrowband 515 nm pulse (~6 cm^−1^ fwhm) by neutralizing the opposite temporal chirp during second harmonic generation in a BBO crystal. A built-in pulse picker can tune the pulse repetition rate up to 200 kHz.

For the homodyne SFG measurement geometry, a narrowband *p*-polarized visible pulse at 515 nm and a broadband *p*-polarized IR pulse centered at 5,150 nm (~100 cm^−1^ FWHM) were focused on the gold film electrode with an angle of incidence of 65° and 40°, respectively. The reflected *p*-polarized SFG signal was collected by Shamrock S500i spectrometer with its combined Newton EMCCD (Andor Technology, Ltd.).

For the phase-sensitive SFG measurement (Nihonyanagi et al., 2013; Shen, 2013), the angle of incidence of the IR pulse at the first sample stage was set to 55°. The reflected SFG, VIS and IR were again focused onto the second sample stage by a concave mirror. Finally, the phase-sensitive SFG signal was imaged into the detector.

### Self-Assembled Monolayer (SAM) Preparation

Polycrystalline gold film electrodes deposited on microscope slide (Au thickness ~100 nm, Substrata Thin Film) were used as substrates for SAM. The gold films were pretreated in an ultrasonic bath in acetone for 5 min, and then in ethanol for 5 min. These steps were repeated once before drying with nitrogen flow. The cleaned gold films were then immersed in 1 mM **ReSS**/**ReS** in N,N-dimethyl-formamide (DMF) for ~48 h to assemble as a monolayer at the gold film surface. Before SFG measurements, the gold films were rinsed and washed with ethanol three times, and then dried in air.

### Computational Methodology

Density functional theory (DFT) and time-dependent density functional theory (TD-DFT) calculations using the Gaussian 2016 software package, revision A.03 (Frisch et al., 2016), were performed to compute optimized structures, frontier orbitals, vibrational frequencies, reduction potentials and UV/Vis spectra. DFT-based SFG spectra were simulated following previously published protocols (Anfuso et al., 2011, 2012; Clark et al., 2016, 2018b; Ge et al., 2016, 2017). Detailed methods are provided in Supplementary Material, including the description of functionals and basis sets.

## Synthesis

### [Re(SSbpy)(CO)_3_Cl] (ReSS)

The SSbpy ligand (30 mg, 0.138 mmol, 1 eq) and [Re(CO)_5_Cl] precursor (50 mg, 0.138 mmol, 1 eq), were added into 10 mL of 1:1 toluene/tetrahydrofuran (THF). The solution was stirred in the dark for 4 h at 40°C. The pale-yellow solution changed to an intense orange color. After cooling, hexane was added to precipitate out the orange complex. It was filtered and washed with hexane and separated over silica with dichloromethane (DCM). The bright orange fraction was collected and recrystallized from DCM/hexane with a yield of 21%. Anal. Calc. for C_13_H_6_N_2_S_2_O_3_ClRe: % C 29.8, % H 1.1, % N 5.3, % S 12.24. Found: % C 29.1, % H 0.9, % N 4.6, % S 10.1. IR (KBr pellets): 3063 (w), 2028 (s), 1,911 (s), 1,628 (w), 1,418 (m), 1,407 (w), 1,262 (w), 1,215 (w), 1,113 (w), 1,060 (w), 819 (w), 811 (w), 804 (w), 753 (w), 721 (w), 643 (w), 631 (w), 539 (w), 477 (w) cm^−1^. ^1^H-NMR spectrum (600 MHz, CD_3_CN) δ 8.89 (2, dd, 2H, J = 5.4, 1.4), 8.06 (4, dd, 2H, J = 8.1, 1.4), 7.56 (3, dd, 2H J = 8.1, 5.4). ^13^C-NMR (150 MHz, CD_3_CN) δ 151.89 (Clark et al., 2018a), 127.82 (Schneider et al., 2016), 138.65 (Kaim, 2011), 135.21 (Clark et al., 2018b), 153.51 (Zhanaidarova et al., 2019). ESI-MS (MeOH, *m/z*): [M – Cl]^+^ 488.94 (100%). UV-vis (MeCN): λ_max_ (nm, ε [L.mol^−1^.cm^−1^]): 285 (8890), 320 (sh), 418 (1760).

### [Re(Sbpy)(CO)_3_Cl] (ReS)

The Sbpy ligand (23.5 mg, 0.126 mmol, 1 eq) and [Re(CO)_5_Cl] precursor (46 mg, 0.126 mmol, 1 eq) were added into 10 mL of 1:1 toluene/THF. The solution was stirred in the dark for 2 h under reflux. The white powdered solution changed to an intense yellow color. After cooling, the bright yellow solid was filtered and washed with toluene and separated over silica with 10:1 DCM/MeOH. The bright yellow fraction was collected and recrystallized from DCM/hexane with a yield of 71%. Anal. Calc. for C_13_H_6_N_2_SO_3_ClRe: % C 31.7, % H 1.2, % N 5.7, % S 6.5. Found: % C 31.7, % H 1.1, % N 5.8, % S 6.6. IR (KBr pellets): 3060 (w), 3,048 (w), 3,034 (w), 2,026 (s), 1,922 (s), 1,900 (s), 1,627 (w), 1,592 (w), 1,567 (w), 1,422 (m), 1,407 (m), 1,385 (w), 1,305 (w), 1,288 (w), 1,226 (w), 1,149 (w), 1,062 (w), 819 (w), 808 (w), 788 (w), 743 (w), 721 (w), 648 (w), 631 (w), 560 (w), 537 (w), 483 (w) cm^−1^. ^1^H-NMR spectrum (600 MHz, CD_3_CN) δ 8.97 (2, dd, 2H, J = 5.1, 0.9), 7.78 (3, dd, 2H, J = 8.3, 5.1), 8.68 (4, dd, 2H J = 8.3, 0.9). ^13^C-NMR (150 MHz, CD_3_CN) δ 150.24 (Clark et al., 2018a), 126.67 (Schneider et al., 2016), 135.90 (Kaim, 2011), 134.83 (Clark et al., 2018b), 153.30 (Zhanaidarova et al., 2019). ESI-MS (MeOH, *m/z*): [M – l]^+^ 456.96 (100%), [M + Cl] 526.90 (100%),. UV-vis (MeCN): λ_max_ (nm, ε [L.mol^−1^.cm^−1^]): 258 (10,930), 296 (6,070), 307 (sh), 373 (1,990).

## Results and Discussion

### Spectroscopic Characterization

As described above, the complex [Re(SSbpy)(CO)_3_Cl] (**ReSS**) was synthesized by mixing the disulfide ligand (**SSbpy**) with the [Re(CO)_5_Cl] precursor. Since the ligand is known to be unstable at high temperatures, the synthesis was carried out under toluene/THF at 40°C to avoid further decomposition, obtaining a 21% yield (Cattaneo et al., 2018). Complex [Re(Sbpy)(CO)_3_Cl] **(ReS)** was synthesized following known procedures (Worl et al., 1991) by refluxing the [Re(CO)_5_Cl] precursor with the monosulfur ligand, and was obtained with >70% yield. Analytic analysis, NMR and ESI mass spectrometry confirmed the identity of the complexes (Figures S1–S9). Further characterization of the compounds by FTIR spectra in both KBr pellets and in CH_3_CN showed the three typical ν_C=O_ stretches of the fac-Re(CO)_3_ block found in similar compounds (Smieja and Kubiak, 2010), namely the higher energy a' (Qiao et al., 2016) symmetric stretch, as well as the low energy symmetric a' (Clark et al., 2018a) and antisymmetric a” stretches (Figure S10). For both complexes, the ν_C=O_ stretches were observed with similar frequencies (Table 1), suggesting that the ligands had little effect on the Re-CO interactions (*vide infra*).

**Table 1:** Spectral and electrochemical data for **ReSS** and **ReS** complexes.

**Complex**	**ν (CO)/cm^−1^^a^**	**λ_max_/nm (ε/M cm^−1^)^b^**	**E1/2/V^c^ Re^I/II^**	**E1/2/V^c^ L^0/-I^ / Re^I/II^**	**λ_em_/nm**	**ϕ_em_^d^**
**ReS**	2,026 1,923 1,904	370 (2010)	1.39	−1.29/−1.73	610	0.02
**ReSS**	2,025 1,923 1,904	418 (1760)	1.39	−0.85/−1.31	660	0.001

a *IR carbonyl stretches in CH_3_CN solution*.

b *Lowest energy UV-vis absorption maximum in CH_3_CN solution*.

c *vs. SCE*.

d *At 295 K*.

The analysis of the UV-Vis spectra of the complexes in MeCN (Figure S11) reveals two low energy transitions at 418 and 370 nm for **ReSS** and **ReS**, respectively, that can be attributed to metal-ligand to ligand charge transfer (MLLCT) transitions (Figure S12; Vlček and Záliš, 2007). The energy of the lowest transition in **ReS** is similar to the one found for [Re(bpy)(CO)_3_Cl], which shows a MLLCT d_π*Re*(*CO*)3*Cl*_-π^*^ transition at 370 nm in MeCN (Worl et al., 1991). Conversely, **ReSS** shows a transition at a considerably lower energy, 418 nm (Δν = 0.385 eV), equivalent to complexes with strong electron-withdrawing substituents at the 4,4'positions of bpy (Zhanaidarova et al., 2019). The MLLCT transitions exhibit solvatochromic shifts when changing the solvent from MeCN to DCM (Figure S11), supporting the charge transfer nature of the transition. TD-DFT calculations confirmed the MLLCT nature of these low energy transitions (Figure S20 and Table S5). Luminescence spectroscopy was also performed on the two complexes (Figure S13). Complex **ReS** has strong luminescence at 610 nm with quantum yields ϕ = 0.02, and **ReSS** a weak luminescence at 660 nm equivalent to what is obtained with the ruthenium series (Hua et al., submitted).

Suitable crystals for structural analysis of the two complexes could not be obtained. Hence, we performed quantum mechanical calculations at the DFT level to further elucidate the structure of the complexes (details of the calculations can be found in Supplementary Material). Optimized structures in acetonitrile are shown in Figure S18 and selected bond distances and angles are summarized in Table S3. As in previous studies of disulfide bipyridine ligands (Benniston et al., 2009, 2012; Hall et al., 2014), the **ReSS** complex contains a twisted bipyridine ring due to the steric influence of the disulfide bridge, with a torsion angle of ~27°. Conversely, the substitution of the disulfide bond by a thiophene ring maintains the planarity of the bipyridine ligand in the **ReS** complex, resembling the structures found in related [Re(bpy)(CO)_3_Cl] complexes (Busby et al., 2004; Blanco Rodríguez et al., 2005; Smieja and Kubiak, 2010; Kumar et al., 2016). Since the fine coupling between the ligands and metal center controls the energy position of the orbitals, and hence the electronic and redox properties of the compounds, these structural differences suggest that the two complexes should differ in their electrochemical properties (*vide infra*).

### Electrochemical Studies

**Figure 1:**
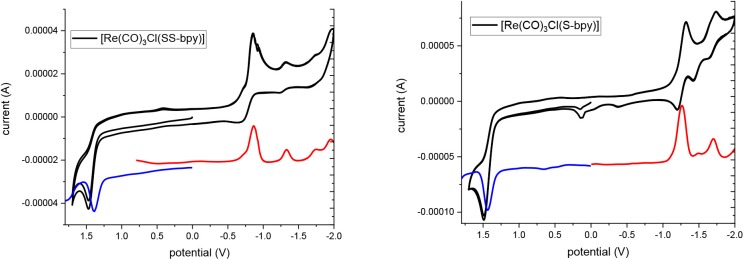
Cyclic voltammograms (black line) and square wave voltammograms (red and blue lines) of complexes in deareated acetonitrile TBAH 0.1 M vs. SCE.

Electrochemical measurements based on cyclic voltammetry (CV), square-wave voltammetry (SWV) and differential pulse voltammetry (DPP) were performed to characterize the redox behavior of these complexes. Figure 1 shows the CV and SWV of **ReSS** and **ReS** with a glassy carbon electrode (GCE). GCE was chosen as it is expected to be inert. At very positive potentials, both complexes exhibit oxidation at 1.39 V vs. SCE, which is close to the oxidation potential of the Re^I/II^ couple for the parent [Re(bpy)(CO)_3_Cl] complex (Worl et al., 1991). At reductive potentials, the two complexes present different behaviors. **ReSS** undergoes two reductions at −0.85 and −1.31 V vs. SCE. The first reduction is reversible when scanning is stopped before the second reduction (Figure S14). The reductive current is consistent with a *two-electron* process. The redox behavior is consistent with dissociative reduction, breaking the disulfide bond (Hall et al., 2014; Cattaneo et al., 2018), which is further supported by spectroelectrochemistry studies and computations as discussed below. The second reduction corresponds to a one-electron reduction of the Re center and is irreversible, likely due to the loss of the chloride ligand (Keith et al., 2013). For the **ReS** complex, the first reduction occurs at 440 mV more negative potential than the **ReSS** compound (−1.29 V vs. SCE) and is attributed to the one-electron reduction of the bipyridine ring (Figure S14; Keith et al., 2013). The second reduction at the Re center is observed at −1.73 V vs. SCE.

**Figure 2:**
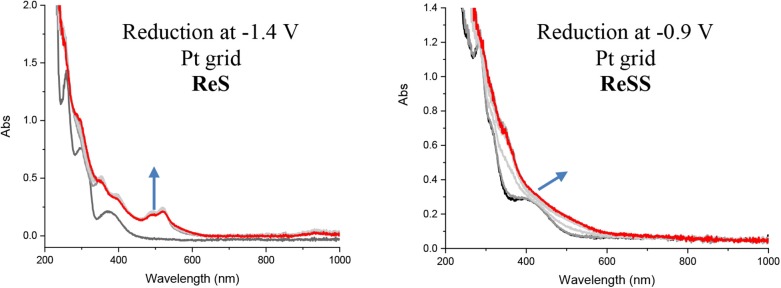
UV-vis spectroelectrochemistry of **ReS** (left) and **ReSS** (right) in deareated MeCN and TBAH (0.1 M) at −1.4 and −0.9 V with Pt grid.

We carried out spectroelectrochemistry of the complexes to further characterize the nature of the species generated by the reductive process. Spectroelectrochemistry was performed both with Pt and Au grid electrodes. Pt was chosen as an inert electrode, while Au is of special interest due to the possibility of immobilizing the complexes on Au. The left panel of Figure 2 (see also Figure S15) shows the transient UV-vis spectra of the **ReS** at the first reduction potential. The spectrum is characterized by the appearance of new bands at 490, 520, and 930 nm, a common observation in polypyridine rhenium complexes (Abate et al., 2018). These bands are consistent with the localization of the electron at the π^*^ orbital of the bipyridine ligand, generating a typical organic radical (Abate et al., 2018). After a second reduction at −1.85 V vs. SCE, there is no effect on the main transitions observed for the radical species (Figure S15). Conversely, when **ReSS** is reduced at −0.9 V in the spectroelectrochemical cell, minimal changes are observed in the MLLCT band (right panel Figure 2, see also Figure S16). There are no new bands characteristic of a radical species up to 1,100 nm. Subsequent reduction at −1.4 V shows the MLLCT band shifting to lower energy without any radical characteristics. These results suggest that a two-electron reduction occurs at the SSbpy ligand to break the disulfide bond, bypassing the radical step. No radical characteristics were observed with either the Pt or Au electrodes, confirming that the reduction of ReSS is a two-electron process independent of the immobilization of the complex. This is in agreement with the CV results and DFT calculations (see below). Similar conclusions can be obtained from the analysis of the changes in the spectroelectrochemistry in the IR carbonyl stretching vibrational region presented in the Supporting Information (Figure S17 and Table S2). For the **ReS** complex, at the potential of the first reduction, the carbonyl stretching shifts c.a. 28 cm^−1^ to lower frequencies. This shift is indicative of the reduction of the bipyridine ligand, and is similar to the one observed for the complex [Re(bpy)(CO)_3_Cl] (Hua et al., submitted). Conversely, after the first reduction of **ReSS** complex at −0.85 V, a very small shift is observed for the carbonyl peaks (Δν = 9 cm^−1^) even though this step comprises a two-electron process. These results reinforce the fact that the first reduction in **ReSS** occurs at the disulfide bridge without the formation of a radical intermediate.

**Figure 3:**
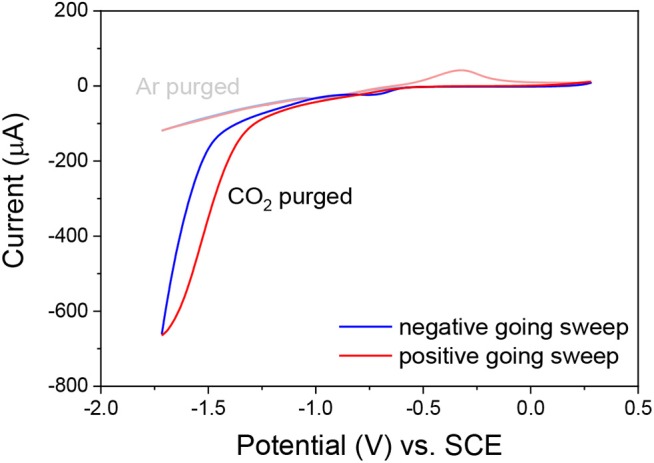
Cyclic voltammograms of complex **ReSS** in acetonitrile TBAH 0.1 M solutions purged with Ar and CO_2_. A catalytic current is observed in the CO_2_-purged solution. WE, gold film; RE, Ag wire; CE, Pt wire. Internal reference Fc.

Cyclic voltammetry of a fresh solution of **ReSS** with a gold film working electrode showed a large current increase when purged with CO_2_ compared to the Ar purged voltammograms (Figure 3), demonstrating that the complex presents catalytic activity. The onset at ~−1.40 V vs. SCE is in agreement with previous studies of these family of complexes, where the reduction at the Re^0/I^ couple makes the complex active for CO_2_ reduction (Clark et al., 2018a). Further studies are needed to fully characterize the catalytic performance of the complexes.

**Figure 4:**
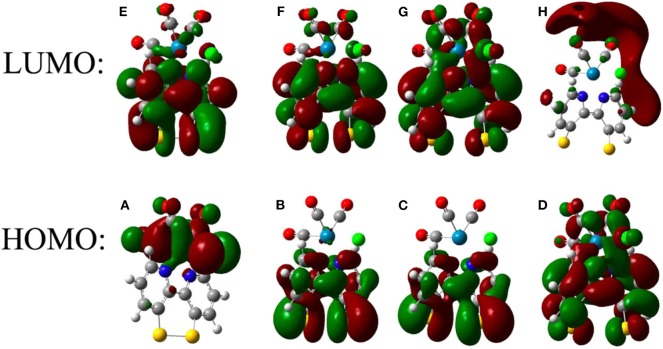
Frontier orbitals of **ReSS** after successive one-electron reductions. The frontier orbitals are shown before reduction **(A,E)** as well as after one **(B,F)**, two **(C,G)**, and three **(D,H)** reductions.

Further insight into the reduction processes of the two complexes was obtained from the computational results. Figure 4 shows the frontier molecular orbitals for the **ReSS** complex after sequential reduction. The molecular contributions to the orbitals were analyzed, revealing that the three lowest unoccupied levels of both complexes are localized on the bpy ligand and sulfide groups (Figure S19 and Table S4). The LUMO of **ReSS** shows a mixture of aromatic and antibonding disulfide character, which promotes cleavage of the S-S bond upon reduction (Hall et al., 2014). Consistently, the frontier orbitals of **ReSS**^**−**^ and **ReSS**^**2−**^ show localized electron density on the S atoms with exclusively antibonding character. The geometrical parameters of the optimized structures (Table S3) also show an increase in the S-S bond distance and an opening of the bipyridine dihedral angle for the reduced species. Unlike the free ligand (Cattaneo et al., 2018) and related disulfide molecules (Llarena et al., 2006; Hall et al., 2014) where the sulfur atoms rotate away from each other with dihedral angles close to ~100°, however, the constraints imposed by the bonding of the bipyridine ligand to the metal center preclude total rotation of the aromatic rings making the ligand suitable for anchoring to gold surfaces. The Re center is not reduced until the third electron reduction (**ReSS**^**3−**^), in agreement with the experimental results that suggest the **ReSS** complex can accept three electrons. Additional insight is provided by the calculated reduction potentials for **ReSS** (Tables S1, S7). Although the absolute value of the reduction potential depends on the level of theory and functional (see Tables S6, S7), the **ReSS**^**−**^ → **ReSS**^**2−**^ reduction is always predicted at lower potentials than the **ReSS** → **ReSS**^**−**^ reduction. These results indicate that the reduction of the disulfide bond is a two-electron process with potential inversion, in accordance with experiments on molecules with a disulfide group coupled to biphenyl or bipyridine rings (Benniston et al., 2009, 2012; Hall et al., 2014; Cattaneo et al., 2018). The free energy for the loss of a Cl^−^ atom is also consistent with experimental observations (Table S8), suggesting loss of the Cl^−^ after the third reduction of **ReSS** forms [Re(SSbpy)(CO)_3_]^2−^. Note that the five-coordinate [Re(L)(CO)_3_]^2−^ is the active species involved in the CO_2_ reduction mechanism (Keith et al., 2013; Clark et al., 2018a).

**Figure 5:**
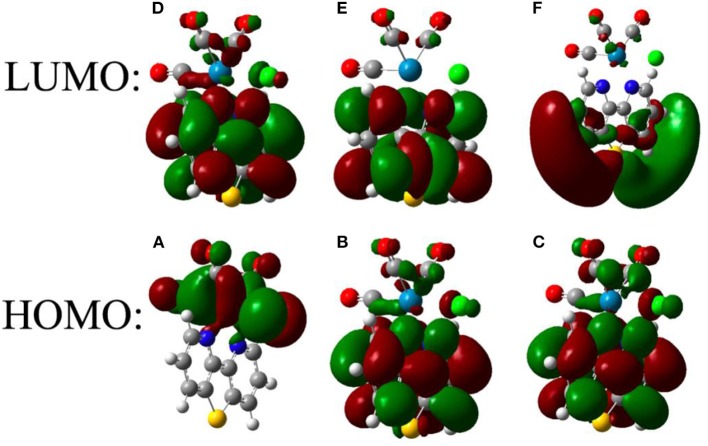
Frontier orbitals of **ReS** after successive one-electron reductions. The frontier orbitals are shown before reduction **(A,D)** as well as after one **(B,E)** and two **(C,F)** reductions.

Figure 5 shows the analysis of the frontier orbitals of **ReS** consistent with the distinct electrochemical behavior of that complex. The first one-electron reduction is mainly localized on the bipyridine ring, with little density on sulfur, while the second reduction is localized on both the bipyridine and the metal center (Keith et al., 2013). The calculated reduction potentials (Tables S1, S7) for **ReS** → **ReS**^**−**^ and **ReS**^**−**^ → **ReS**^**2−**^ are well-separated and in good agreement with experimental data.

### Sum Frequency Generation Spectroscopy

**Figure 6:**
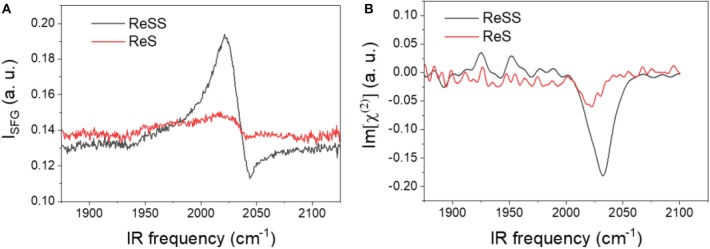
Homodyne **(A)** and heterodyne **(B)** SFG spectra of **ReSS** and **ReS** SAMs on gold.

Self-assembled monolayers (SAM) of **ReS** and **ReSS** on Au films were probed by SFG to characterize the synthesized molecular catalyst as heterogenized on gold surfaces, with an emphasis on characterization of the binding motif of the complexes on the surface. SFG is a surface-selective technique with submonolayer sensitivity (Nihonyanagi et al., 2013; Shen, 2013; Ge et al., 2019). When combined with computational modeling, it can provide information on molecular orientation at the interface. Figure 6 shows the homodyne and heterodyne SFG spectra in the CO stretching region for both complexes. For the **ReS** monolayer, no discernable peak can be determined within significant signal-to-noise ratio. The absence of signal for this complex is indicative of the lack of adsorption. For **ReSS** monolayers, a strong peak at ~2,030 cm^−1^ is observed that can be attributed to the totally symmetric a' (Qiao et al., 2016) stretching mode of the Re(CO)_3_ moiety. Within signal-to-noise, no peaks for the out-of-phase symmetric and asymmetric stretching modes can be accurately determined. These results suggest strong binding of the **ReSS** complex to the gold surface.

**Figure 7:**
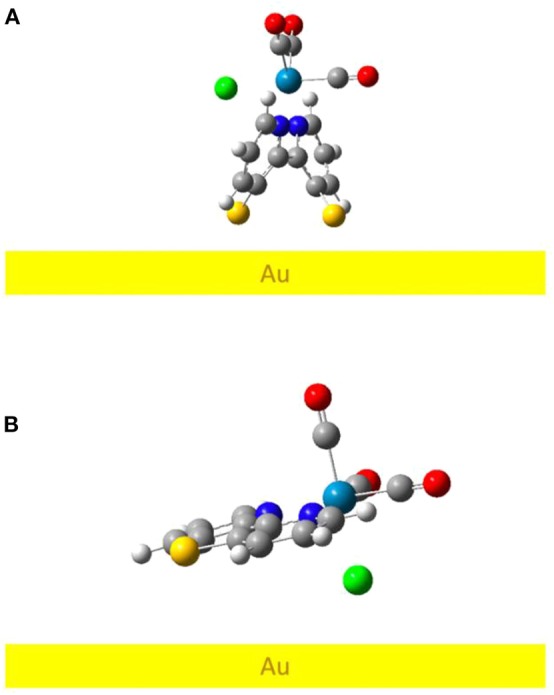
DFT-optimized geometry for **(A) ReSS** and **(B) ReS** complexes on gold slab.

We performed DFT optimizations of the complex on an Au (111) model surface to better understand the binding conformation of the Re complex adsorbed on the gold electrode as compared to previously reported studies of Re complexes on surfaces (Anfuso et al., 2011, 2012; Clark et al., 2016, 2018b; Ge et al., 2016, 2017). Figure 7 shows the lowest-energy minima geometry for both **ReSS** and **ReS** complexes (see also Figure S21). The optimized structure of **ReSS** consists of an almost “standing-up” configuration with both sulfur atoms anchored to the gold surface, with sulfur-gold distances of ~2.6 Å. Such a perpendicular orientation with respect to the surface contrasts with the geometry adopted by the Re complex with a 4-S-2,2′-bipyridine ligand that adopts a tilted orientation unfavorable for substrate binding (Clark et al., 2018b). Instead, the binding mode of **ReSS** should facilitate CO_2_ binding since the active site is left exposed to the solvent instead of being blocked and strongly interacting with the electrode surface. In contrast, the **ReS** complex does not form a stable sulfur-gold bond (S-Au distance of ~3.6 Å) and remains with a tilted conformation physically absorbed on the gold surface. Note that the absence of preferential binding is consistent with the lack of SFG signal as reported for this complex (*vide supra*).

**Figure 8:**
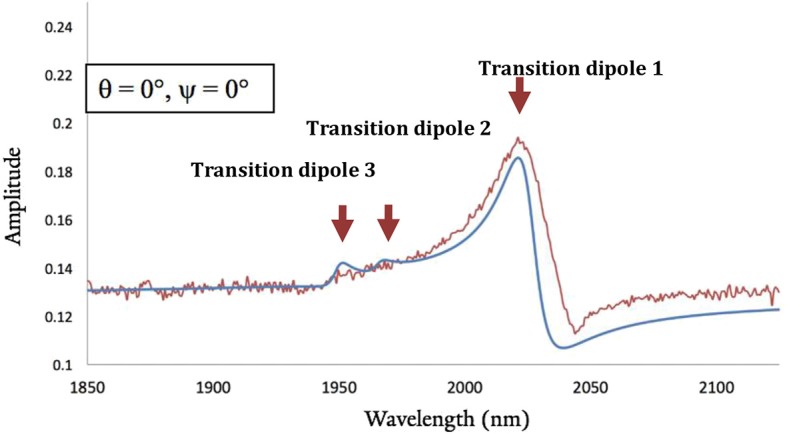
Best-matched DFT-based calculated SFG spectra (blue line) and experimental results (red line) for the homodyne spectra of **ReSS**. θ and ψ correspond to Euler angles that characterized the orientation of the complex with respect to the surface (see supporting information). The orientation with θ = ψ = 0° corresponds to the “standing-up” conformation shown in Figure 7.

We computed the DFT-based homodyne SFG spectra of the complex for a series of orientations to have direct comparisons with the experimental spectrum (see Supplementary Material for additional details) and corroborate that the DFT minimum energy structure of the complexes bound to gold corresponds to the experimentally-observed orientation on the surface (Anfuso et al., 2011, 2012; Clark et al., 2016, 2018b; Ge et al., 2016, 2017). We focused on **ReSS** for which we have reliable experimental SFG spectra. Figure 8 shows the comparison of calculated and experimental spectra (fitting parameters are provided in Table S9). The agreement between theory and experiments is satisfactory, supporting the predicted orientation of the complex on the gold surface. Note that the perpendicular orientation is the only one in which the high frequency carbonyl stretch dominates the spectrum and the low frequency carbonyl stretches are suppressed (Figures S22, S23). In this “standing up” configuration, the halogen atom is exposed to the solution, providing a favorable orientation for redox state transitions and catalysis. Further, the bidentate binding mode should enable the complex to remain bound at more strongly reducing potentials than the previously reported 4-S-2,2′-bipyridyl Re complex, which desorbed at −1.3 V of applied potential (Clark et al., 2018b). Future work will characterize the catalytic and spectroscopic behavior of the surface-attached complex in *operando* conditions.

## Conclusions

We have introduced two chloro-tricarbonyl rhenium complexes with thio-substituted bipyridine ligands for CO_2_ reduction, [Re(SSbpy)(CO)_3_Cl] (**ReSS**) and [Re(Sbpy)(CO)_3_Cl] (**ReS**) that were characterized as homogenous and heterogenized systems using spectroscopic, computational and electrochemical techniques. The two complexes have significantly different reduction potentials and electronic transitions. In particular, **ReSS** presents an electronic transition 0.385 eV lower in energy than **ReS**, typical of strong electron withdrawing bipyridyl ligands. The first reduction of **ReSS** is observed at −0.85 V (vs. SCE) and involves a two-electron process with no evidence of radical formation, consistent with reduction of the disulfide bridge in a two-electron process. DFT calculations show that the first reduction requires a higher potential than the second reduction of the complex (potential inversion), and that both reductions involve antibonding orbitals localized in the disulfide bond. Conversely, **ReS** behaves similarly to other Re bipyridyl complexes, with the first one-electron reduction forming a radical species localized in the bipyridine ring, and the second, irreversible, one-electron reduction localized on the Re center. The third reduction of ReSS produces a catalytic current at ~−1.40 V (vs. SCE) under CO_2_ atmosphere on a gold electrode.

We have probed the complexes heterogenized on gold electrodes by using SFG spectroscopy. The **ReSS** catalyst exhibits a strong SFG signal consistent with robust binding to the gold surface. DFT calculations of the SFG spectrum and direct comparisons to the experimental spectra show that the **ReSS** complex adopts a minimum energy configuration with both thiolate groups formed upon reduction of the disulfide bridge covalently bound to the gold surface and the bpy ligand oriented perpendicular to the surface. The halogen ligand is exposed to the solvent, with a favorable orientation for exchange with the CO_2_ substrate. The bidentate binding mode should be more resistant to reducing potentials than previously reported monodentate-binding complexes, and is thus particularly promising for a wide range of applications, including complexes based on other metal centers, such as Mn. The capabilities of the SSbpy non-innocent ligand for storage of multiple reducing equivalents and the favorable orientation of the **ReSS** complex attached to gold electrodes makes this novel catalyst an interesting system for future *in-operando* studies of CO_2_ reduction.

## Data Availability Statement

The datasets analyzed in this article are not publicly available. Requests to access the datasets should be directed to Tianquan Lian, tlian@emory.edu; Mauricio Cattaneo, mcattaneo@fbqf.unt.edu.ar; Víctor S. Batista, victor.batista@yale.edu.

## Author Contributions

MC, TL, and VB contributed conception and design of the study. MC wrote the first draft of the manuscript. FG, HK, PV, LK, SG, AG, QL, and SW wrote sections of the manuscript. All authors contributed to manuscript revision, read, and approved the submitted version.

### Conflict of Interest

The authors declare that the research was conducted in the absence of any commercial or financial relationships that could be construed as a potential conflict of interest.
