# Supplementary material for: Robust Binding of Disulfide-Substituted Rhenium Bipyridyl Complexes for CO2 Reduction on Gold Electrodes
Source: Front Chem. 2020 Feb 13;8:86. doi: 10.3389/fchem.2020.00086 (PMC7031654; doi:10.3389/fchem.2020.00086)
Supplement: Supplementary file 1 [file Data_Sheet_1.PDF]

Supplementary Material

Robust Binding of Disulfide-Substituted Rhenium Bipyridyl Complexes for CO_2 Reduction on Gold Electrodes

Mauricio Cattaneo[1]*, Facheng Guo[2], H. Ray Kelly[2], Pablo E. Videla[2], Laura Kiefer[3], Sara Gebre[3], Aimin Ge[3], Qiliang Liu[3], Shaoxiong Wu[3], Tianquan Lian[3]* and Víctor S. Batista[2]*

[1] INQUINOA-UNT-CONICET, Instituto de Química Física, Fac. Bioquímica, Química y Farmacia, Universidad Nacional de Tucumán, Ayacucho 471, T4000INI San Miguel de Tucumán, Argentina
[2] Department of Chemistry, Yale University, New Haven, Connecticut 06520-8107, United States
[3] Department of Chemistry, Emory University, Atlanta, Georgia 30322, United States

1. ESI MASS SPECTROMETRY 2
2. NMR 2
3. FTIR 7
4. UV-VIS 7
5. LUMINESCENCE 9
6. ELECTROCHEMISTRY 9
7. SPECTROELECTROCHEMISTRY 10
8. COMPUTATIONAL 14
9. REFERENCES 21

1. ESI Mass Spectrometry

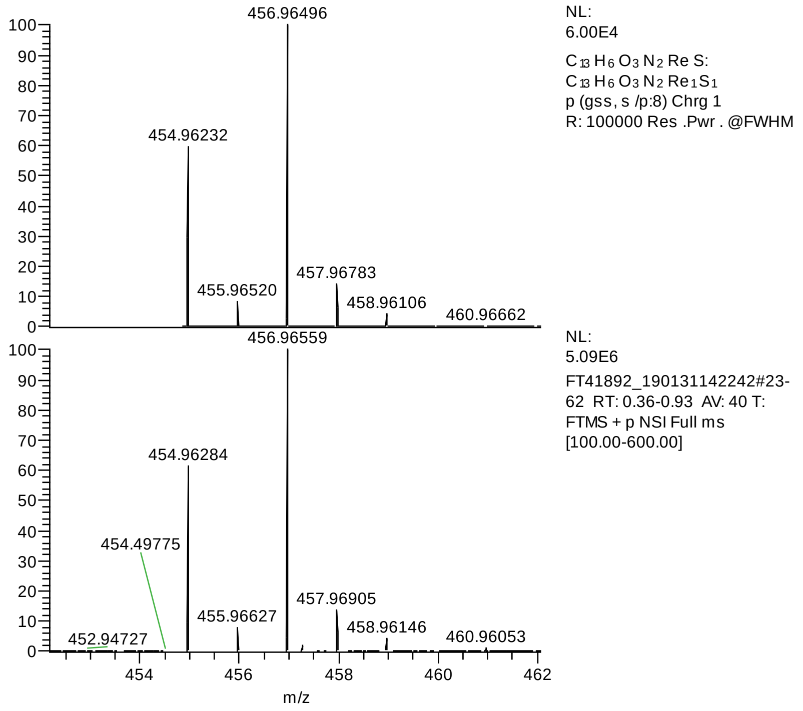

FIGURE S1. Experimental and simulated isotopic pattern from ESI-HRMS of main peak **ReS**+.

2. NMR

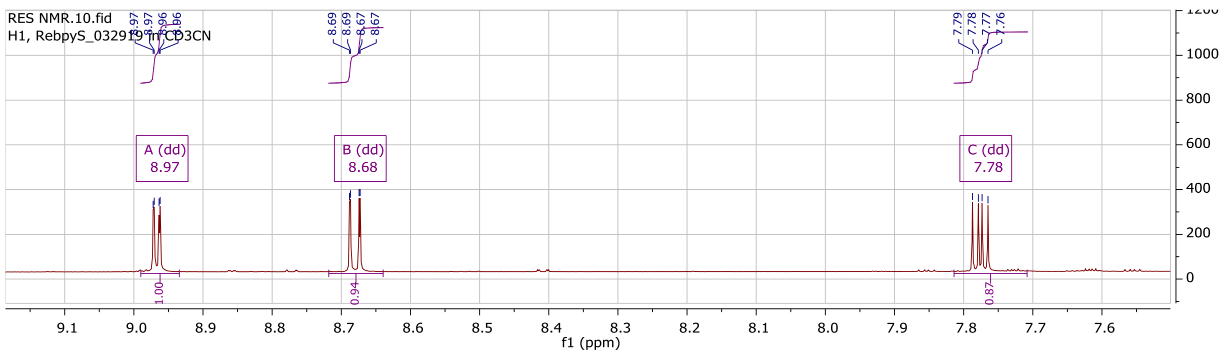

FIGURE S2. ^{1}H-NMR in acetonitrile-d_3 of **ReS**.

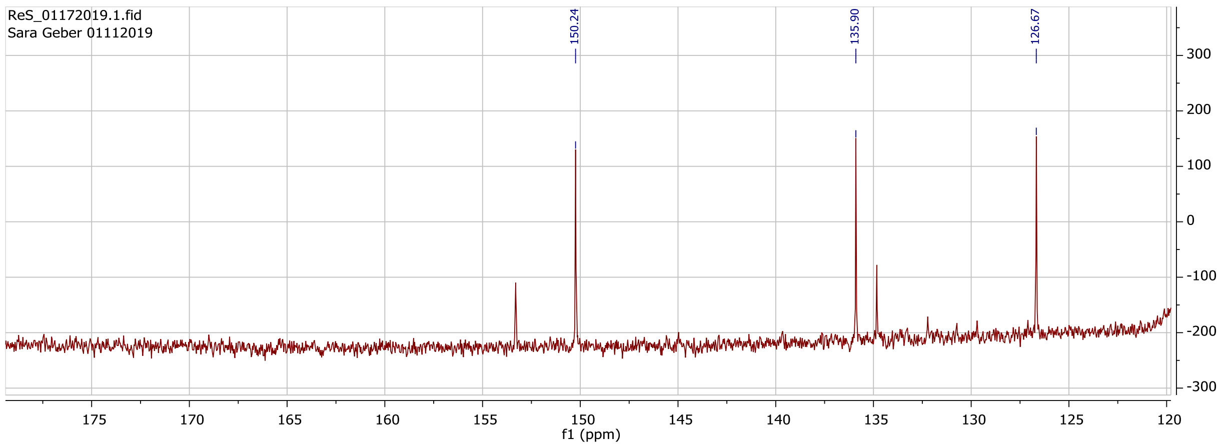

FIGURE S3. ^{13}C-NMR in acetonitrile-d_3 of **ReS**.

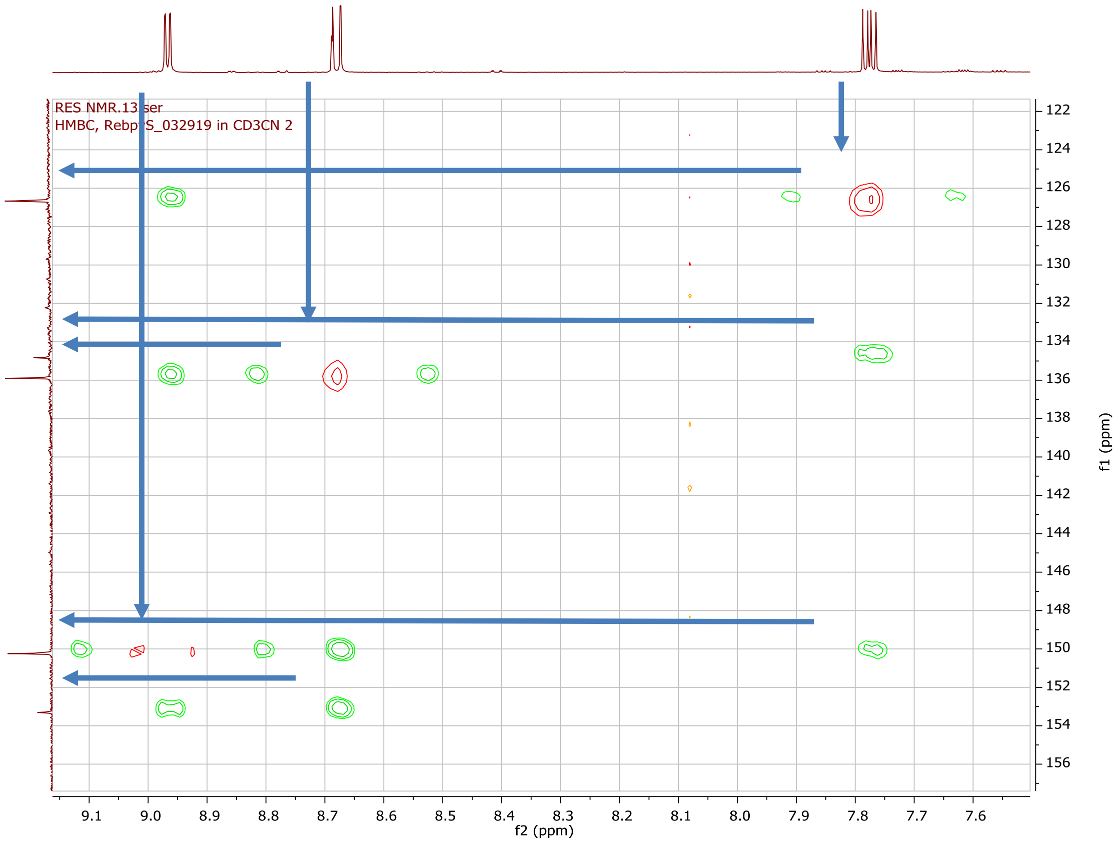

FIGURE S4. ^{1}H-NMR, ^{13}C-NMR and ^{1}H-^{13}C-HSQC-HMBC in acetonitrile-d_3 of **ReS**.

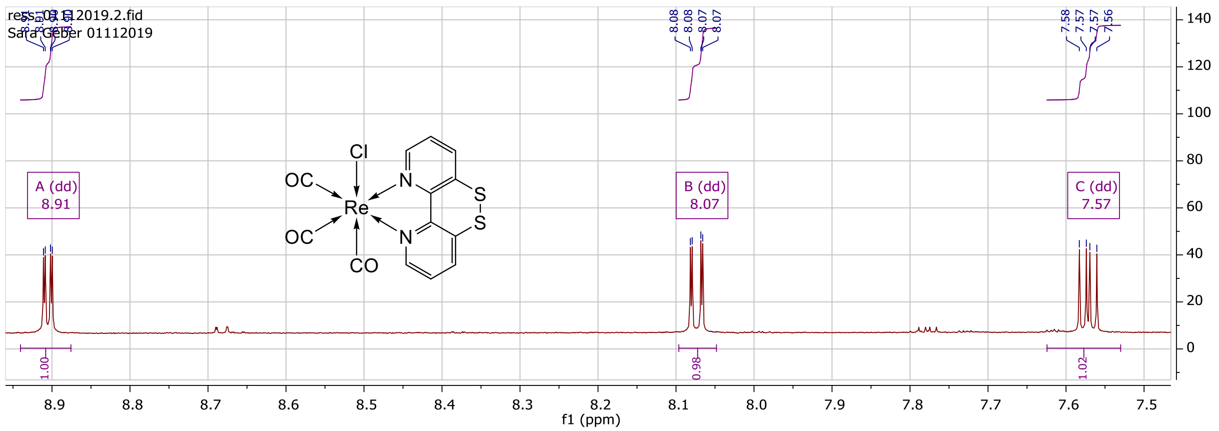

FIGURE S5. ^{1}H-NMR in acetonitrile-d_3 of **ReSS**.

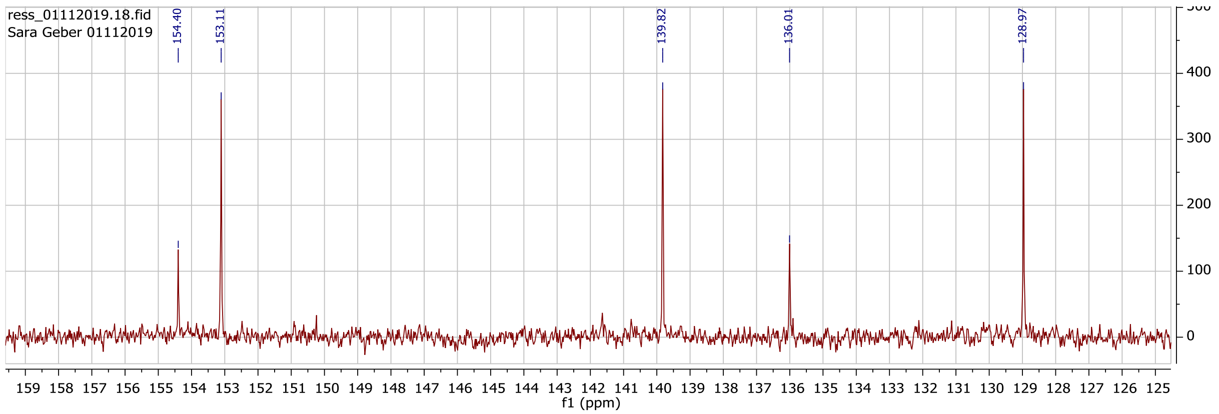

FIGURE S6. ^{13}C-NMR in acetonitrile-d_3 of **ReSS**.

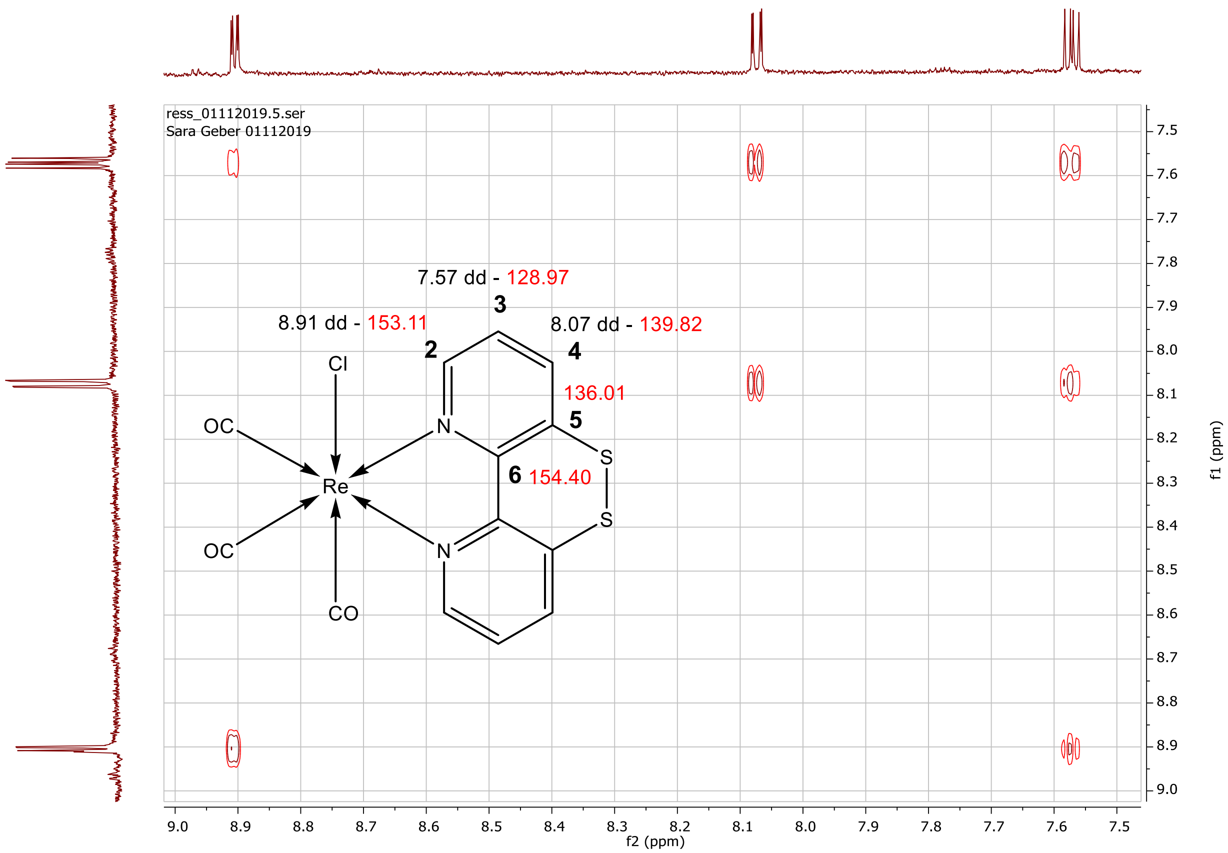

FIGURE S7. $^{1}H^{1}H$-COSY in acetonitrile-d_3 of **ReSS**.

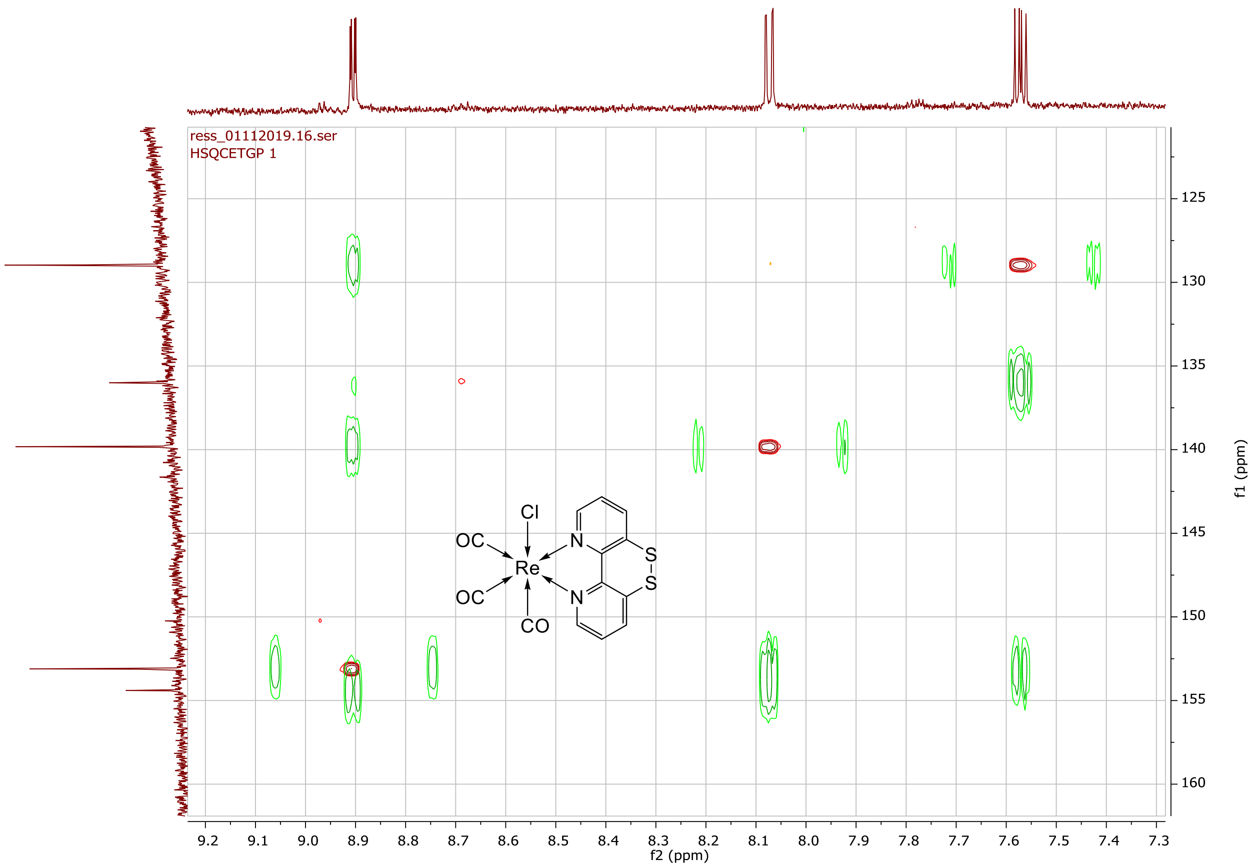

FIGURE S8. $^1H^1H$-COSY, $^1H^{13}C$-HSQC and $^1H^{13}C$-HMBC in acetonitrile-d_3 of **ReSS**.

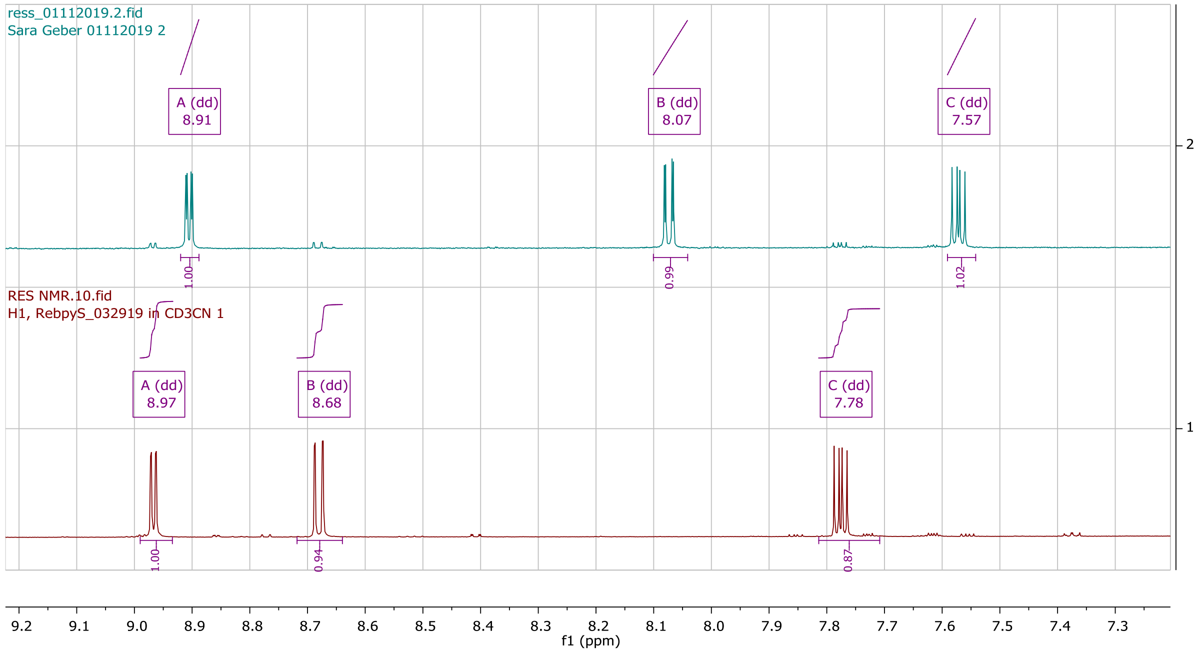

FIGURE S9. ^1H-NMR in acetonitrile-d_3 of complexes.

3. FTIR

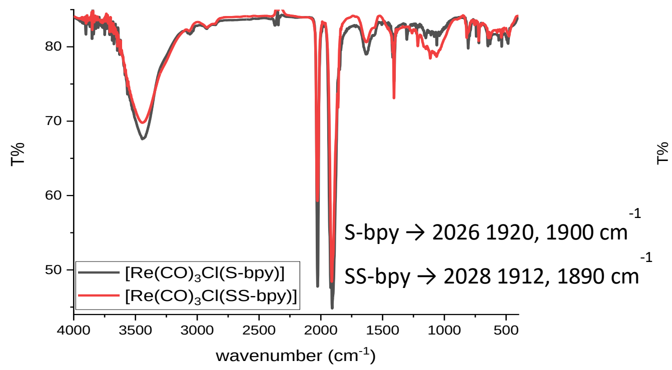

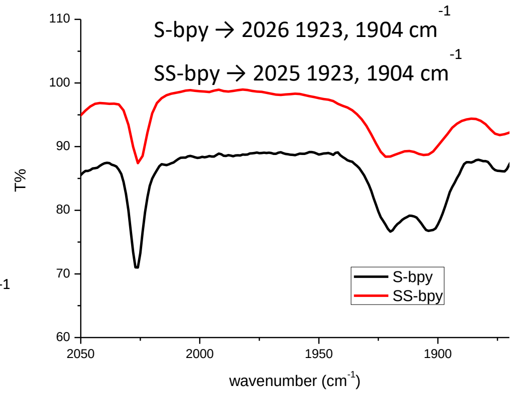

FIGURE S10. FTIR spectra in KBr pellets (left) and MeCN solutions (right) of complexes.

4. UV-Vis

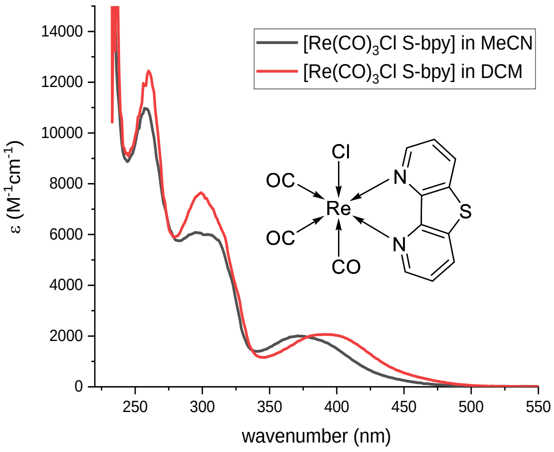

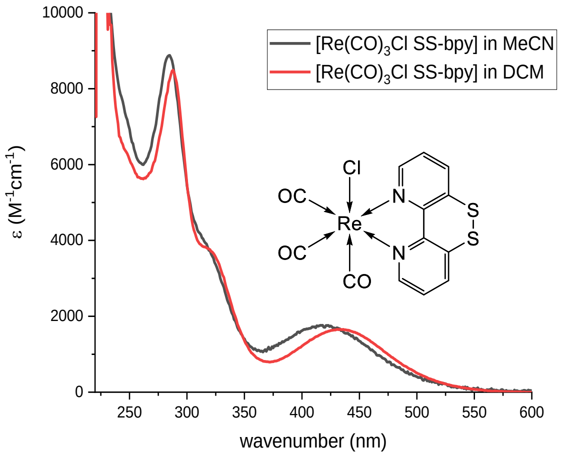

FIGURE S11. UV-Vis spectra of **ReS** (left) and **ReSS** (right) complexes in MeCN and DCM.

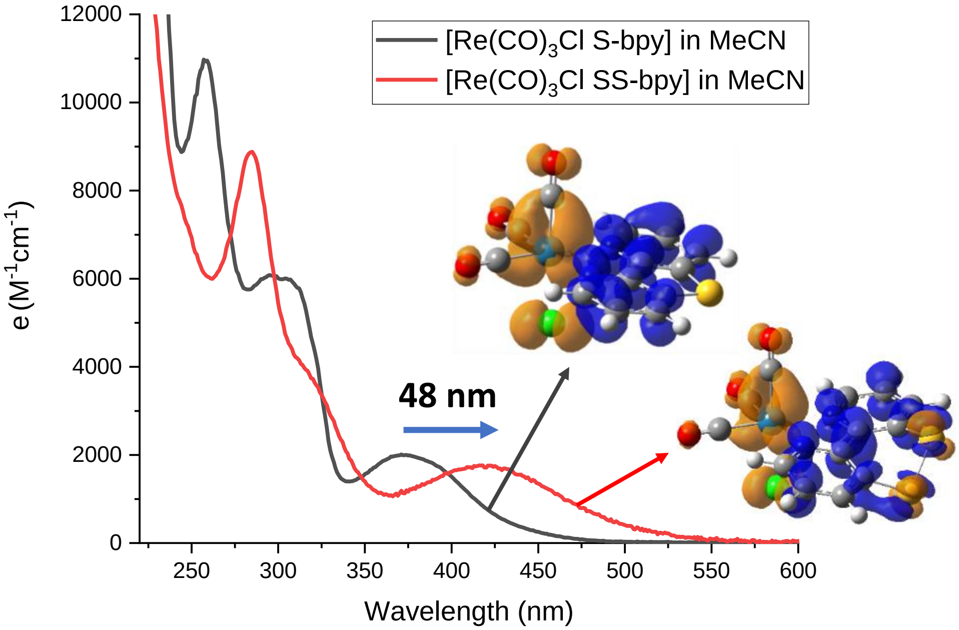

FIGURE S12. UV-vis spectra in acetonitrile of **ReSS** (grey) and **ReS** (red). Inset plot: Electron density difference map (EDDM) for lowest MLLCT transitions. Orange: decrease in electron density; Blue: increase in electron density.

5. Luminescence

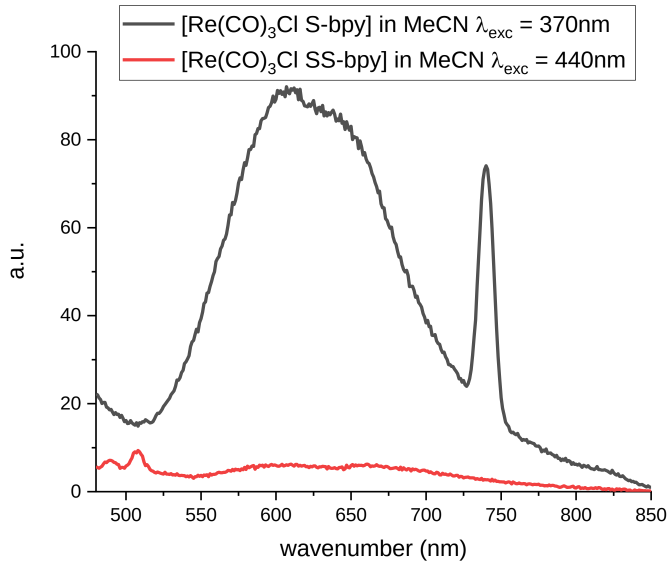

FIGURE S13. Luminescence of **ReS** and **ReSS** in deareated acetonitrile.

6. Electrochemistry

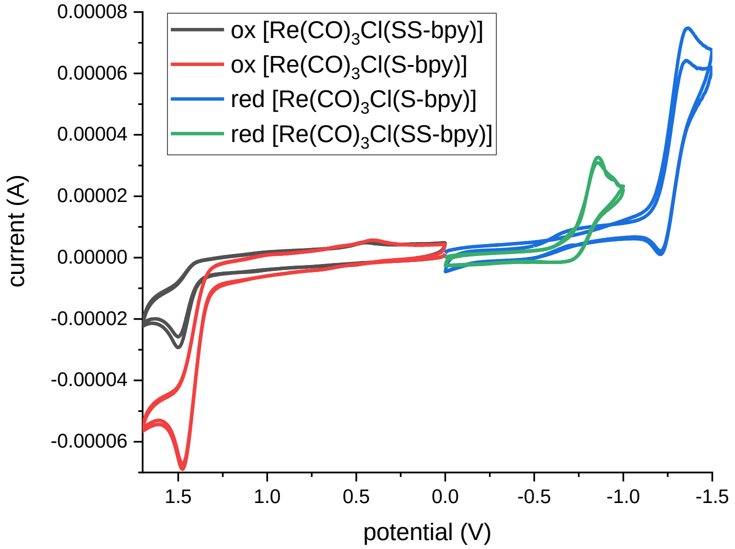

FIGURE S14. CV for first reduction and oxidation of complexes in deareated acetonitrile TBAH 0.1 M vs SCE.

TABLE S1. Experimental and computed values for oxidation and reduction potentials (in V, vs. SCE).

Complex	$E_{½}$ $Re_{I/II}$	$E_{½}$ $L_{0/-I}$ / $Re_{I/II}$	$E_{½}$ $L_{0/-I}$ / $Re_{I/II}$
	Experimental		*Computed*
$[Re(bpy)(CO)_3Cl]$	1.38	-1.35[a]<br>-1.40/-1.80[d]<br>-1.34/-1.73[c]	-1.23/-1.76[b]
$[Re(S\text{-}bpy)(CO)_3Cl]$	1.39	-1.29/-1.73	-1.20/-1.95(-1.67[e])
$[Re(SS\text{-}bpy)(CO)_3Cl]$	1.39	-0.85/-1.31	-0.52[g]/-2.07(-1.61[e],-1.12[f])

[a] from Worl's work[1]. [b] from Riplinger's work[2]. [c] from Smieja's work[3]. [d] from Clark's work[4]. [e] including an H^+. [f] including two H^+. [g] two-electron process.

7. Spectroelectrochemistry

TABLE S2. Experimental and calculated ν_{CO} frequencies (in cm^{-1}) of both complexes and reduced species by spectroelectrochemistry.

	ν_{CO} (Vacuum) (DFT)[a]	ν_{CO} (Acetonitrile) (DFT)[b]	ν_{CO} (Acetonitrile) (Exp.)
$[Re(S\text{-}bpy)(CO)_3Cl]$	2004<br>1928<br>1902	2040<br>1922<br>1906	2026<br>1923<br>1904
$[Re(S\text{-}bpy)(CO)_3Cl]^{-1}$	1972<br>1880<br>1862	2019<br>1893<br>1879	1998<br>1886<br>1860
$[Re(S\text{-}bpy)(CO)_3Cl]^{-2}$	1925<br>1825<br>1816	1975<br>1852<br>1842	1996-1986-1975<br>1884-1874-1862-1856
$[Re(SS\text{-}bpy)(CO)_3Cl]$	2001<br>1927<br>1904	2036<br>1923<br>1907	2025<br>1923<br>1904
$[Re(SS\text{-}bpy)(CO)_3Cl]^{-2}$	1977<br>1888<br>1873	2030<br>1909<br>1894	2014<br>1902<br>1884
$[Re(SS\text{-}bpy)(CO)_3Cl]^{-3}$	1956<br>1853<br>1848	2025<br>1900<br>1886	2008 (1996-1987)<br>1875

[a]Frequencies are scaled by 0.96. [b]Frequencies are scaled by 0.99.

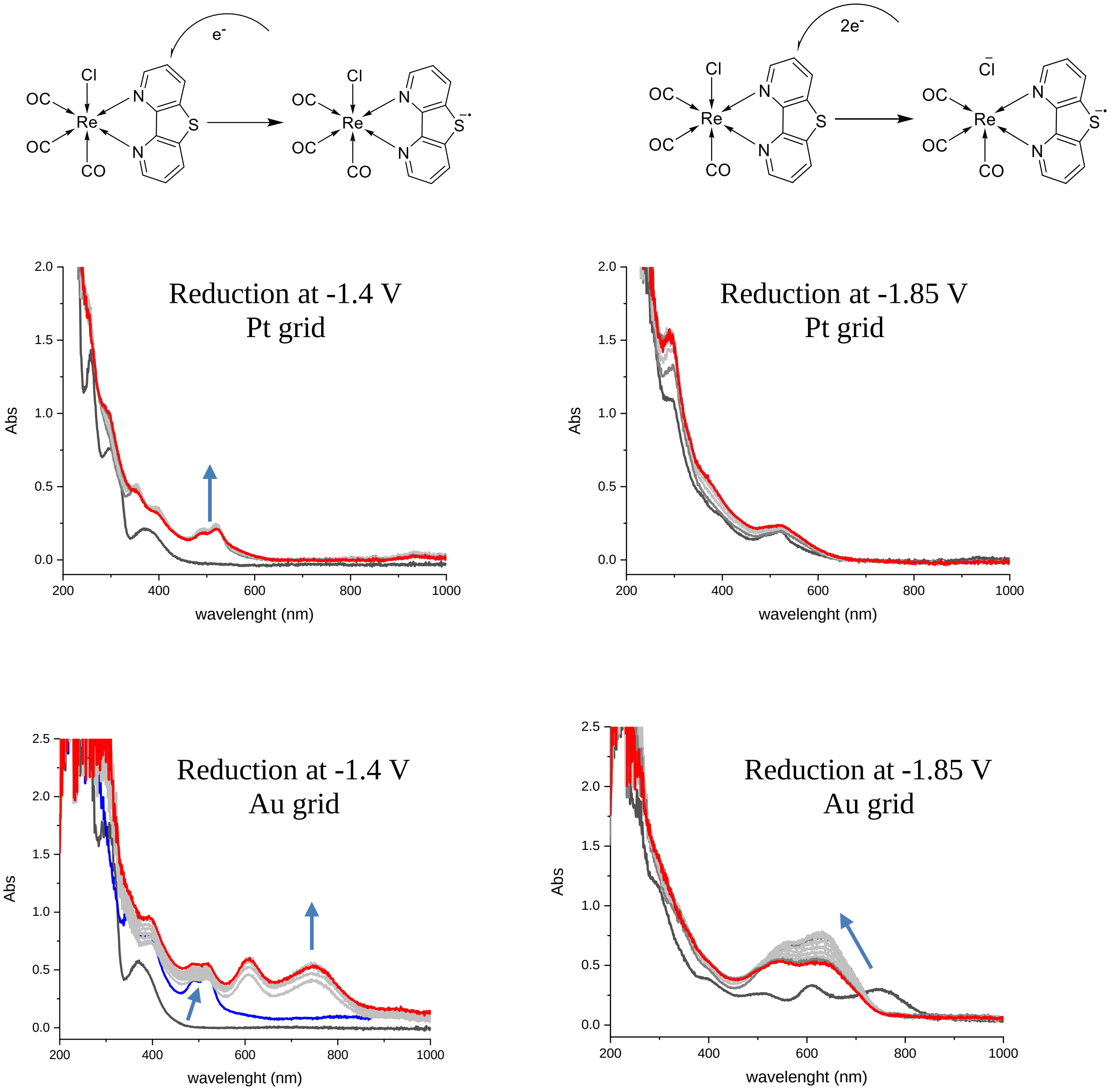

FIGURE S15. UV-Vis spectroelectrochemistry of **ReS** in (Pt and Au)-OTTLE cell in a MeCN/0.1M TBAH mixture, at -1.4V and -1.85 V.

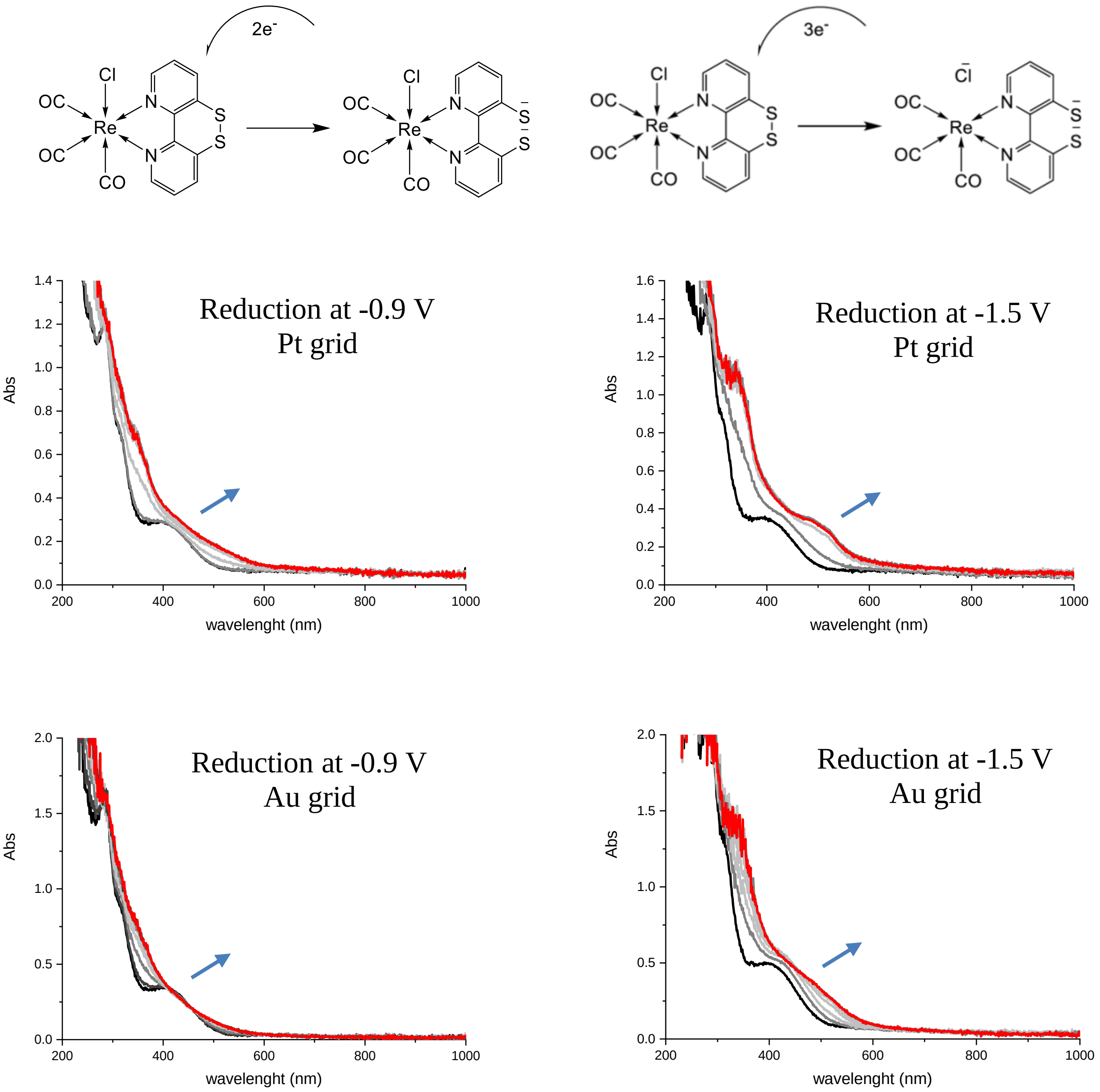

FIGURE S16. UV-Vis spectroelectrochemistry of **ReSS** in (Pt and Au)-OTTLE cell with acetonitrile TBAH 0.1 M, at -0.9V and -1.5 V.

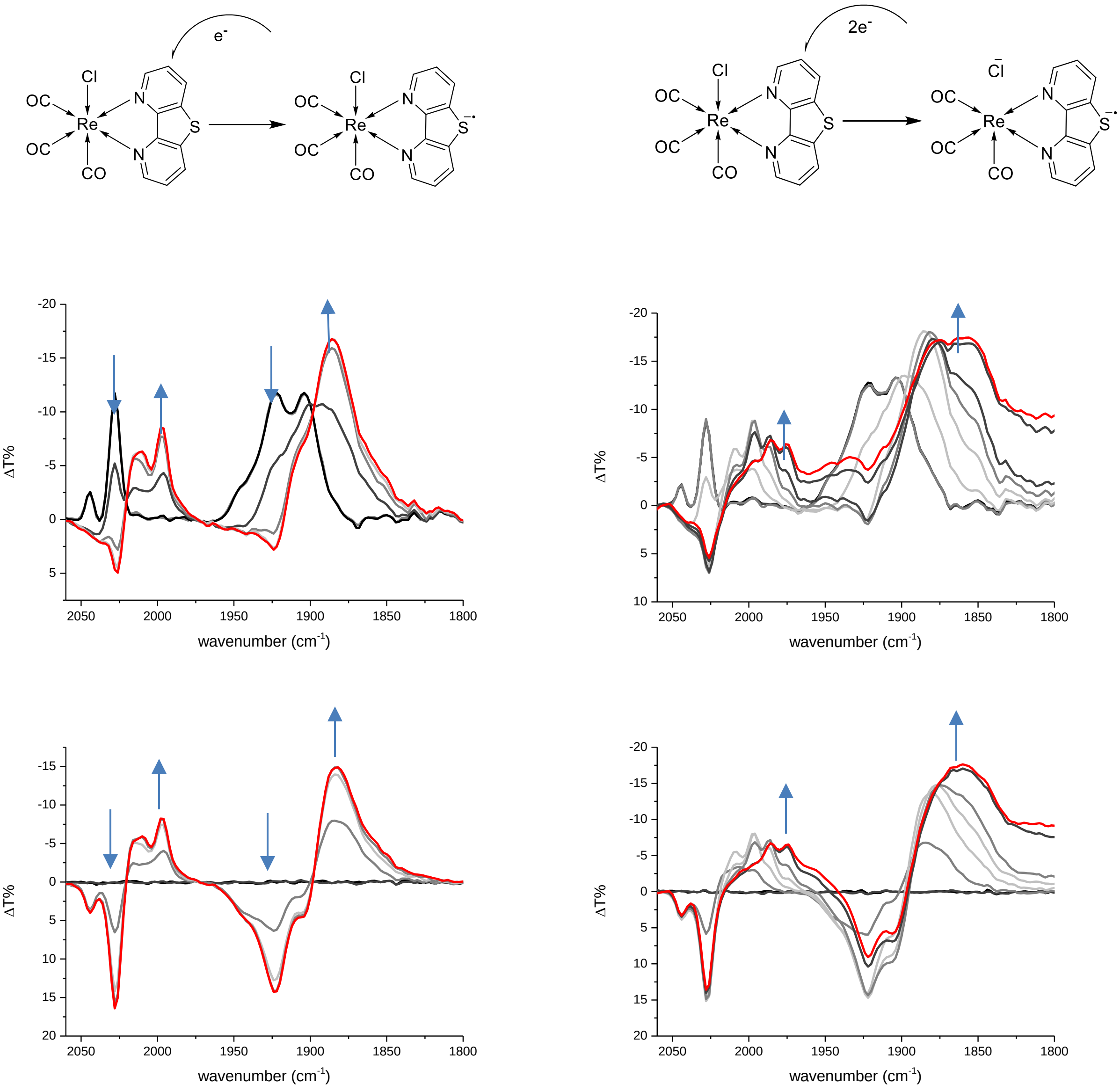

FIGURE S17. FTIR spectroelectrochemistry of **ReS** in OTTLE cell in an MeCN/0.1 M TBAH mixture, at -1.3V (left) and -1.6 V (right) vs SCE.

8. Computational

Infrared and UV/Vis spectra. Density functional theory (DFT) calculations were performed using the Gaussian 2016 software package, revision A.03[5]. Geometry optimizations were performed using the (U)B3LYP functional[6, 7] with the 6-31+G(2df,p) basis sets[8, 9] on all non-metallic atoms and the def2TZVP effective core potential and basis sets[10, 11] on the Re atom. Solvation effects were included by employing a solvation model based on density (SMD[12]) for acetonitrile. Vibrational frequency calculations were performed to characterize the stationary structures and compute the CO stretching frequencies (Table S2), employing scaling factors of 0.96 and 0.99 for vacuum and acetonitrile calculations (scaling factors were determined as average ratio between the neutral experimental and theoretical result). Optimized structures of both complexes in acetonitrile are shown in Figure S18 and selected bond distances and angles are summarized in Table S3. Frontier orbitals (Figure S19, Table S4) were visualized using GaussView. UV-Vis spectra was based on vacuum optimized structure and were computed by time-dependent DFT (TD-DFT) calculations employing 60 states and the (U)B3LYP functional, 6-311+G(2df,p) basis sets[13-23] on all non-metallic atoms and the def2TZVP effective core potential and basis sets[10, 11] on the Re atom with SMD of acetonitrile (Figure S21, Table S5).

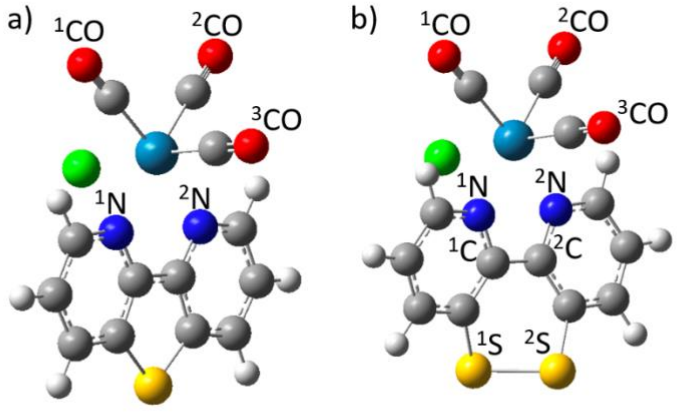

FIGURE S18. Optimized structures for **ReS** (left) and **ReSS** (right) complexes in acetonitrile.

TABLE S3. Structural information for **ReS** and **ReSS** complexes.[a]

	**ReS**	**ReS^{-}**	**ReS^{2-}**	**ReSS**	**ReSS^{-}**	**Re-SS^{2-}**	**Re-SS^{3-}**
Re-Cl (Å)	2.50	2.54	2.66	2.50	2.52	2.55	2.69
Re-^{1}CO (Å)	1.93	1.93	1.93	1.94	1.93	1.93	1.93
Re-^{2}CO (Å)	1.93	1.93	1.93	1.94	1.93	1.93	1.93
Re-^{3}CO (Å)	1.93	1.92	1.89	1.93	1.92	1.92	1.89
Re-^{1}N (Å)	2.28	2.26	2.22	2.23	2.22	2.22	2.19
Re-^{2}N (Å)	2.28	2.26	2.22	2.22	2.22	2.22	2.19
^{1}S-^{2}S (Å)	---	---	---	2.07	2.49	3.36	3.50
^{1}S^{1}C-^{2}C^{2}S (°)	---	---	---	26.8	34.4	55.4	56.3

[a]See Figure S18 for labeling.

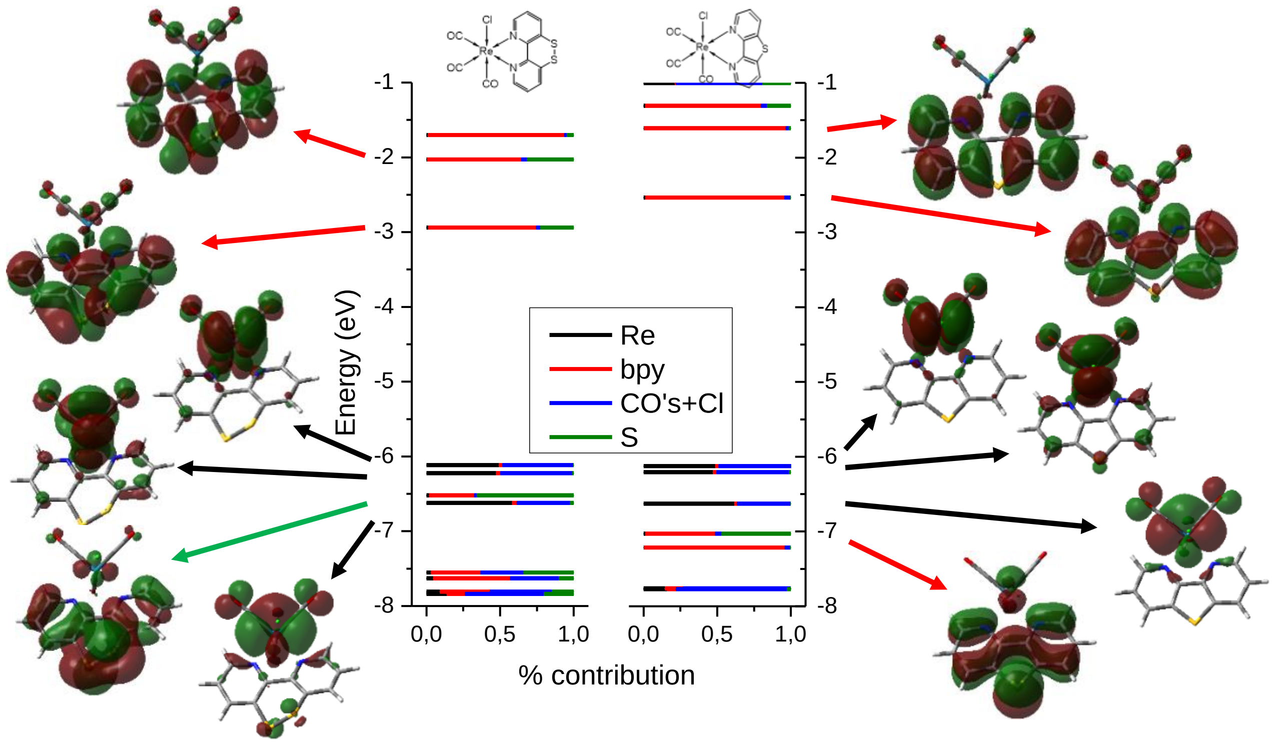

FIGURE S19. Frontier MO's for **ReS** (left) and **ReSS** (right) with percentage contribution from different groups in the molecule (– black line for Re, – red line for bipyridyl ligand, – blue line for Cl and carbonyls, and – green line for S).

TABLE S4. Frontier MO's for **ReS** and **ReSS** with percentage contribution from different groups in the molecule PDOS.

MO	Energy (eV)	**Re**	**SSbpy**	**Cl+CO**	**SS**	**Re**	**Sbpy**	**Cl+CO**	**S**	Energy (eV)	MO
L+4	-0.90	17	12	56	15	19	9	71	1	-0.78	L+4
L+3	-0.96	5	6	14	75	21	1	59	19	-1.00	L+3
L+2	-1.70	1	**93**	2	5	1	**78**	4	16	-1.31	L+2
L+1	-2.03	1	**64**	4	**32**	0	**96**	2	1	-1.61	L+1
LUMO	-2.94	1	**73**	3	**23**	1	**94**	4	0	-2.53	LUMO
HOMO	-6.12	**49**	2	**48**	0	**49**	2	**49**	0	-6.13	HOMO
H-1	-6.22	**47**	3	**49**	1	**47**	2	**49**	0	-6.21	H-1
H-2	-6.52	2	**31**	2	**66**	**62**	2	**36**	0	-6.63	H-2
H-3	-6.62	**58**	3	**36**	2	1	**47**	4	**47**	-7.03	H-3
H-4	-7.55	3	**34**	**29**	**34**	0	**96**	3	1	-7.22	H-4

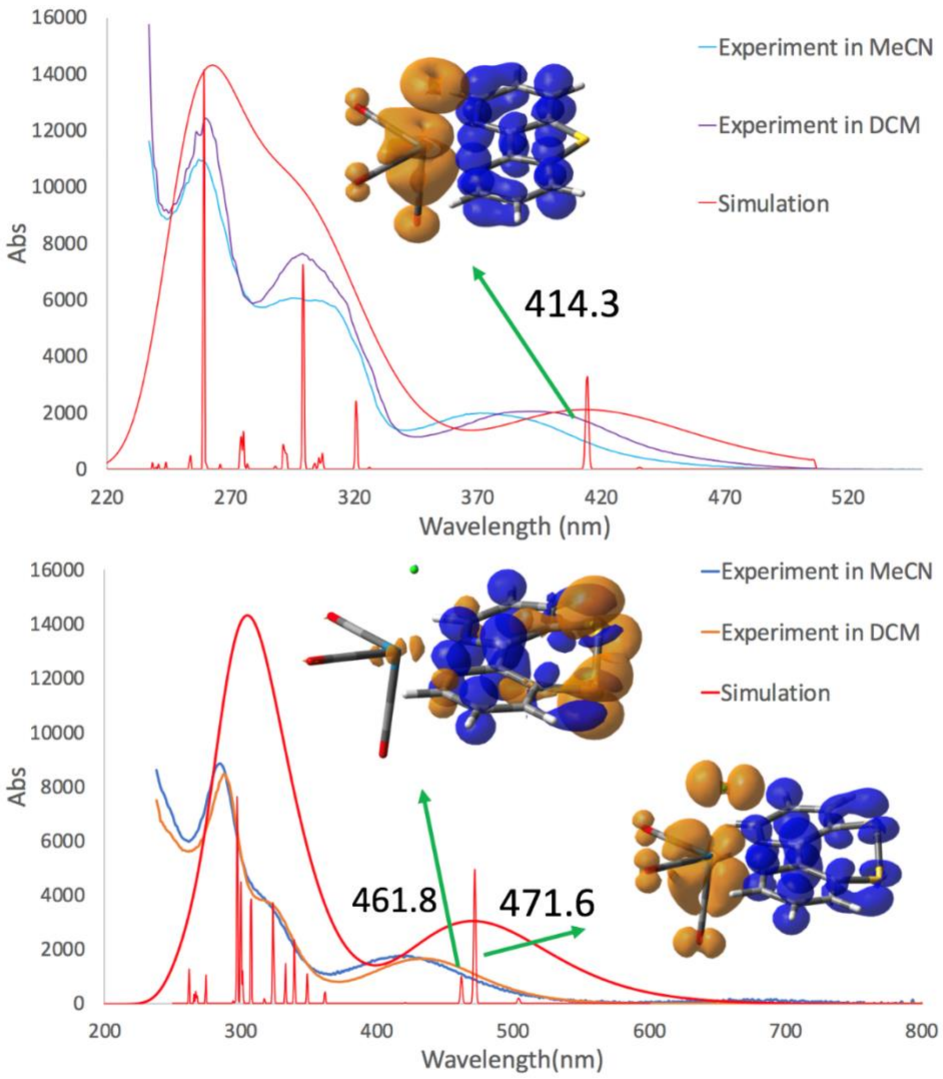

FIGURE S20. Simulated UV-Vis spectra and electron density difference map (EDDM) for lowest MLLCT transitions in **ReS** (top panel) and **ReSS** (bottom panel). Orange: decrease in electron density; blue: increase in electron density.

TABLE S5. Lowest MLLCT transitions for **ReS** and **ReSS** with percentage contribution from different groups in the transitions.

Complex	λ_{max} (nm)	Osc. Sth.	transition	Re	bpy	Cl+CO's	S/SS
**ReS**	414.29	0.0643	H-1->LUMO (100%)	47-->1 (-46)	2-->94 (92)	49-->4 (-45)	1-->0 (-1)
**ReSS**	471.57	0.0614	H-1->LUMO (96%)	46-->1 (-45)	3-->73 (70)	48-->3 (-45)	2-->23 (21)
	461.80	0.0122	H-2->LUMO (92%)	4-->1 (-3)	30-->72 (42)	3-->3 (0)	63-->23 (-40)

Reduction potentials. Reduction potentials were computed from the Gibbs free energy difference obtained at the DFT level. The calculations included geometry optimizations in gas phase using a medium-sized basis set. Thermochemical energy contributions were included by using the ideal gas, rigid rotor, and harmonic oscillator approximations at a temperature of 298.15 K. Electronic energies at the optimized geometries were computed using a larger basis sets. Different combinations of functional and basis sets were employed to assess the error in the reduction potential (Tables S6 and S7). In all calculations, the D3 Grimme's empirical dispersion[24] was used. Solvation energies (from geometries optimized in acetonitrile with the medium-sized basis sets) were computed at the larger basis sets by employing the polarizable continuum model (PCM[25]). All reported reduction potential values are referenced with respect to the SCE, taking the absolute potential of SCE in acetonitrile to be -4.422 V[2].

TABLE S6. Different basis sets used to compute the reduction potential of **ReS** and **ReSS**.

	Basis sets #1	Basis sets #2	Basis sets #3	Basis sets #4
Functional	(U)B3LYP	(U)B3LYP	(U)B3LYP	(U)WB97XD
Optimization/Frequency calculation	6-31+G(2df,p): C N S H LANL2DZ: Re Cl	6-31+G(2df,p): C N Cl S H Def2SVP: Re	Def2SVP: C N Cl S H Re	6-31+G(2df,p): C N S H LANL2DZ: Re Cl
Single point calculation	AUG-cc-pVTZ: C N S H LANL2DZ: Re Cl	6-31+G(2df,p): C N Cl S H Def2SVP: Re	Def2SVP: C N Cl S H Re	AUG-cc-pVTZ: C N S H LANL2DZ: Re Cl

TABLE S7. Computed one-electron reduction potentials (V vs. SCE) of **ReS** and **ReSS**, for different basis sets combinations.[a]

Reduction	Basis sets #1	Basis sets #2	Basis sets #3	Basis sets #4
**ReS** → **ReS^{-}**	-1.20	-1.39	-1.38	-1.31
**ReS^{-}** → **ReS^{2-}**	-1.95 (-1.67[b])	-2.14	-2.40	-1.94
**ReSS** → **$ReSS^{-}$**	-0.61	-0.90	-0.77	-0.92
**$ReSS^{-}$** → **$ReSS^{2-}$**	-0.42	-0.76	-0.91	-0.21
**$ReSS^{2-}$** → **$ReSS^{3-}$**	-2.07 (-1.61[c]/-1.12[d])	-2.28	-2.61	-2.15

[a]See Table S6 for definition. [b]For **ReS^{-}** + H^{+} → **ReS^{2-}** + H^{+} reduction. [c]For **$ReSS^{2-}$** + H^{+}→ **$ReSS^{3-}$** + H^{+} reduction. [d]**$ReSS^{2-}$** + $2H^{+}$→ **$ReSS^{3-}$** + $2H^{+}$ reduction.

TABLE S8. Gibbs Free Energy for $[Re(S\text{-}bpy)(CO)_3Cl]^{-n}$ → $[Re(S\text{-}bpy)(CO)_3Cl]^{-n+1}$ + Cl^- and $[Re(SS\text{-}bpy)(CO)_3Cl]$ → $[Re(SS\text{-}bpy)(CO)_3Cl]^{-n+1}$ + Cl^- reactions.[a]

Molecule	Charge (n)	ΔG (kcal/mol)
**$[Re(S\text{-}bpy)(CO)_3Cl]$**	0	3.01
	1	-4.37[b]
	2	-20.00
**$[Re(SS\text{-}bpy)(CO)_3Cl]$**	0	3.71
	1	0.25
	2	10.85
	3	-14.71

[a] Calculation performed using the (U)B3LYP functional[6, 7] with the 6-31+G(2df,p) basis ses[8, 9] on all non-metallic atoms and the def2TZVP effective core potential and basis set[10, 11] on the Re atom, with SMD[12] acetonitrile solvent. [b] The energy barrier is 7.3 kcal/mol, estimated by doing a relaxed potential energy scan of Re-Cl distance.

SFG Simulation. A model gold slab was obtained from periodic boundary conditions DFT calculations in a two-step procedure, using the Vienna ab initio Simulation Package (VASP[26-29]). First, a bulk lattice relaxation was performed using the Perdew, Burke, and Ernzerhof (PBE) generalized gradient exchange-correlation functional[30, 31], the projector augmented plane wave (PAW) method[32, 33], and the D3 dispersion function with Becke Johnson damping [24, 34]. An energy cutoff of 500 eV, energy convergence criterion per unit cell of 10^{-6} eV and a 12×12×12 Monkhorst−Pack k-point grid was used, along with a smear parameter of 0.1 eV and the method of Methfessel-Paxton of order 1[35]. From the optimized bulk structure, a gold slab of 6×6×4 atoms is generated and optimized using a Monkhorst-Pack k-point grid of 1×1×1 and ~40 Å of vacuum. Only the top two layers of the gold slab are alloweds to relax in this calculation. The two uppermost gold layers are used as a model gold slab in the subsequent SFG calculations.

Simulations of SFG spectra for the **ReSS** complex on gold are computed using Gaussian 2016 software package, following our previous work[36-38]. Briefly, a neutral **ReSS** molecule is optimized on the gold slab (kept frozen) using the PBE functional (Geometry A in Table S22) or PW91PW91 functional[39] (Geometry B, C, D and E in Table S22) and the 6-31G(d) basis sets[13, 14, 40-47] for C, H, N, Cl, O, S elements and the LANL2DZ basis sets[48-51] and pseudopotential for Re and Au atoms. Frequencies and hyperpolarizabilities are computed and used to calculate the SFG spectra as in previous work[36-38]. To better characterize the orientation of the complex on the surface, we utilized the Euler angles θ, ψ and φ to relate the molecular frame to the laboratory frame fixed on the surface (see Figure S23). Here, θ is the tilt angle of the bipyridine plane relative to the surface normal, φ is the azimuthal rotation of the molecule relative to the surface normal, and ψ is the twist rotation of the complex along the c axis.

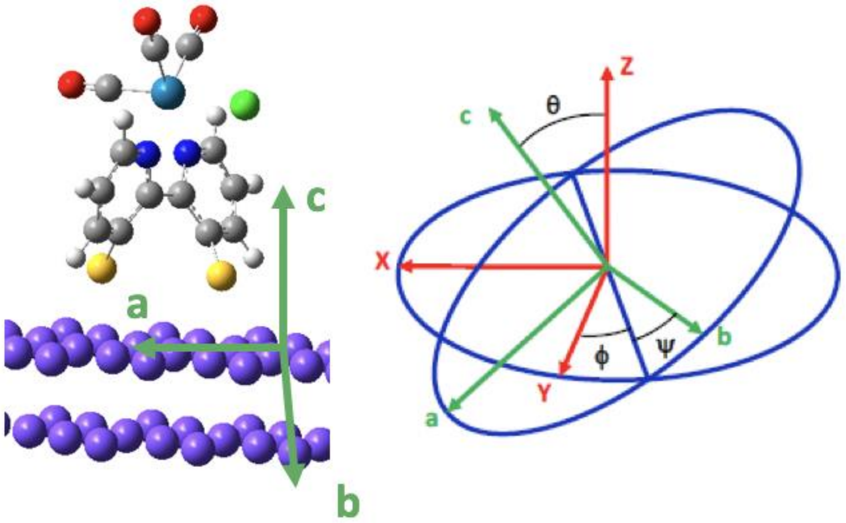

FIGURE S21. Left: Structure of the Re catalyst with the molecular frame axes *a* (aligned with Au-Au bond), *b* (in the gold slab plane and perpendicular with a), and *c* (perpendicular with the gold slab plane) labelled. Right: Euler angle definitions. Color code for atoms: yellow = S, green = Cl, gray = C, blue = N, red = O, azure = Re, white = H, violet = Au.

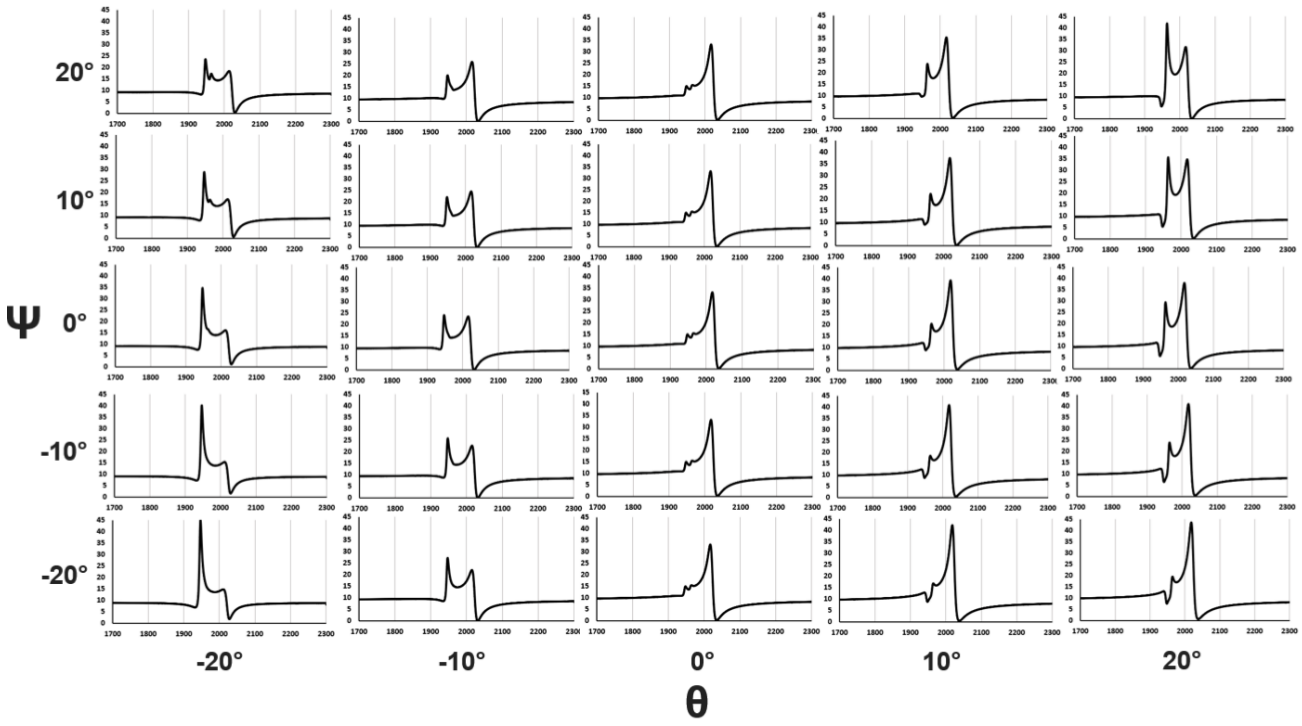

FIGURE S22. DFT-based calculated SFG spectra (black line) with different combination of Ψ and θ angles for the homodyne spectra of **ReSS**.

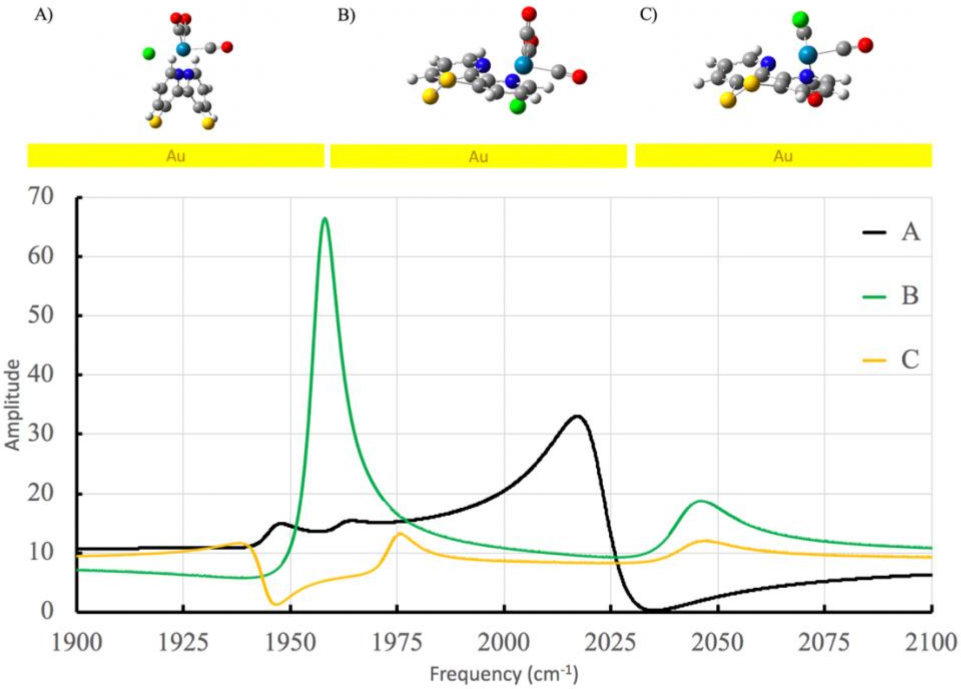

FIGURE S23. Different optimized geometries of **ReSS** on gold slab and corresponding SFG spectra. A) The standing up geometry. B) The Cl facing down geometry. C) The Cl facing up geometry.

TABLE S9. Fitting parameters for the best-matched DFT-based SFG spectra shown in Figure 7.[a]

Molecule	Mode	$X_{eff,q}$	$\omega_q(cm^{-1})$	$\Gamma_q(cm^{-1})$
**[Re(SS-bpy)(CO)$_3$Cl]**	1	49.9	2022	4.0
	2	-1.02	1962	4.0
	3	-2.11	1946	8.0

[a] For all calculations A_{NR} is 3.0, phase is 140°.

9. References

1. Worl, L.A., Duesing, R., Chen, P., Ciana, L.D., Meyer, T.J., Photophysical properties of polypyridyl carbonyl complexes of rhenium(I). Journal of the Chemical Society, Dalton Transactions 1991, (S), 849-858.
2. Riplinger, C., Sampson, M.D., Ritzmann, A.M., Kubiak, C.P., Carter, E.A., Mechanistic Contrasts between Manganese and Rhenium Bipyridine Electrocatalysts for the Reduction of Carbon Dioxide. Journal of the American Chemical Society 2014, 136(46), 16285-16298.
3. Smieja, J.M., Kubiak, C.P., Re(bipy-tBu)(CO)3Cl−improved Catalytic Activity for Reduction of Carbon Dioxide: IR-Spectroelectrochemical and Mechanistic Studies. Inorganic Chemistry 2010, 49(20), 9283-9289.
4. Clark, M.L., Rudshteyn, B., Ge, A., Chabolla, S.A., Machan, C.W., Psciuk, B.T., Song, J., Canzi, G., Lian, T., Batista, V.S., Kubiak, C.P., Orientation of Cyano-Substituted Bipyridine Re(I) fac-Tricarbonyl Electrocatalysts Bound to Conducting Au Surfaces. The Journal of Physical Chemistry C 2016, 120(3), 1657-1665.
5. Gaussian 09 Revision D.01, F., M. J.; Trucks, G. W.; Schlegel, H. B.; Scuseria, G. E.; Robb, M. A.; Cheeseman, J. R.; Scalmani, G.; Barone, V.; Mennucci, B.; Petersson, G. A.; Nakatsuji, H.; Caricato, M.; Li, X.; Hratchian, H. P.; Izmaylov, A. F.; Bloino, J.; Zheng, G.; Sonnenberg, J. L.; Hada, M.; Ehara, M.; Toyota, K.; Fukuda, R.; Hasegawa, J.; Ishida, M.; Nakajima, T.; Honda, Y.; Kitao, O.; Nakai, H.; Vreven, T.; Montgomery, J. A., Jr.; Peralta, J. E.; Ogliaro, F.; Bearpark, M.; Heyd, J. J.; Brothers, E.; Kudin, K. N.; Staroverov, V. N.; Kobayashi, R.; Normand, J.; Raghavachari, K.; Rendell, A.; Burant, J. C.; Iyengar, S. S.; Tomasi, J.; Cossi, M.; Rega, N.; Millam, N. J.; Klene, M.; Knox, J. E.; Cross, J. B.; Bakken, V.; Adamo, C.; Jaramillo, J.; Gomperts, R.; Stratmann, R. E.; Yazyev, O.; Austin, A. J.; Cammi, R.; Pomelli, C.; Ochterski, J. W.; Martin, R. L.; Morokuma, K.; Zakrzewski, V. G.; Voth, G. A.; Salvador, P.; Dannenberg, J. J.; Dapprich, S.; Daniels, A. D.; Farkas, Ö.; Foresman, J. B.; Ortiz, J. V.; Cioslowski, J.; Fox, D. J. Gaussian, Inc., Wallingford CT, 2009.
6. Kim, K., Jordan, K.D., Comparison of Density Functional and MP2 Calculations on the Water Monomer and Dimer. The Journal of Physical Chemistry 1994, 98(40), 10089-10094.
7. Stephens, P.J., Devlin, F.J., Chabalowski, C.F., Frisch, M.J., Ab Initio Calculation of Vibrational Absorption and Circular Dichroism Spectra Using Density Functional Force Fields. The Journal of Physical Chemistry 1994, 98(45), 11623-11627.
8. Petersson, G.A., Al-Laham, M.A., A complete basis set model chemistry. II. Open-shell systems and the total energies of the first-row atoms. The Journal of Chemical Physics 1991, 94(9), 6081-6090.
9. Petersson, G.A., Bennett, A., Tensfeldt, T.G., Al-Laham, M.A., Shirley, W.A., Mantzaris, J., A complete basis set model chemistry. I. The total energies of closed-shell atoms and hydrides of the first-row elements. The Journal of Chemical Physics 1988, 89(4), 2193-2218.
10. Weigend, F., Accurate Coulomb-fitting basis sets for H to Rn. Physical Chemistry Chemical Physics 2006, 8(9), 1057-1065.
11. Weigend, F., Ahlrichs, R., Balanced basis sets of split valence, triple zeta valence and quadruple zeta valence quality for H to Rn: Design and assessment of accuracy. Physical Chemistry Chemical Physics 2005, 7(18), 3297-3305.
12. Marenich, A.V., Cramer, C.J., Truhlar, D.G., Universal Solvation Model Based on Solute Electron Density and on a Continuum Model of the Solvent Defined by the Bulk Dielectric Constant and Atomic Surface Tensions. The Journal of Physical Chemistry B 2009, 113(18), 6378-6396.

13. Binning Jr, R.C., Curtiss, L.A., Compact contracted basis sets for third-row atoms: Ga–Kr. Journal of Computational Chemistry 1990, 11(10), 1206-1216.
14. Blaudeau, J.-P., McGrath, M.P., Curtiss, L.A., Radom, L., Extension of Gaussian-2 (G2) theory to molecules containing third-row atoms K and Ca. The Journal of Chemical Physics 1997, 107(13), 5016-5021.
15. Curtiss, L.A., McGrath, M.P., Blaudeau, J.P., Davis, N.E., Binning, R.C., Radom, L., Extension of Gaussian-2 theory to molecules containing third-row atoms Ga–Kr. The Journal of Chemical Physics 1995, 103(14), 6104-6113.
16. Hay, P.J., Gaussian basis sets for molecular calculations. The representation of 3d orbitals in transition-metal atoms. The Journal of Chemical Physics 1977, 66(10), 4377-4384.
17. Krishnan, R., Binkley, J.S., Seeger, R., Pople, J.A., Self-consistent molecular orbital methods. XX. A basis set for correlated wave functions. The Journal of Chemical Physics 1980, 72(1), 650-654.
18. McGrath, M.P., Radom, L., Extension of Gaussian-1 (G1) theory to bromine-containing molecules. The Journal of Chemical Physics 1991, 94(1), 511-516.
19. McLean, A.D., Chandler, G.S., Contracted Gaussian basis sets for molecular calculations. I. Second row atoms, Z=11–18. The Journal of Chemical Physics 1980, 72(10), 5639-5648.
20. Raghavachari, K., Trucks, G.W., Highly correlated systems. Excitation energies of first row transition metals Sc–Cu. The Journal of Chemical Physics 1989, 91(2), 1062-1065.
21. Wachters, A.J.H., Gaussian Basis Set for Molecular Wavefunctions Containing Third-Row Atoms. The Journal of Chemical Physics 1970, 52(3), 1033-1036.
22. Clark, T., Chandrasekhar, J., Spitznagel, G.W., Schleyer, P.V.R., Efficient diffuse function-augmented basis sets for anion calculations. III. The 3-21+G basis set for first-row elements, Li–F. Journal of Computational Chemistry 1983, 4(3), 294-301.
23. Frisch, M.J., Pople, J.A., Binkley, J.S., Self-consistent molecular orbital methods 25. Supplementary functions for Gaussian basis sets. The Journal of Chemical Physics 1984, 80(7), 3265-3269.
24. Grimme, S., Antony, J., Ehrlich, S., Krieg, H., A consistent and accurate ab initio parametrization of density functional dispersion correction (DFT-D) for the 94 elements H-Pu. The Journal of Chemical Physics 2010, 132(15), 154104.
25. Scalmani, G., Frisch, M.J., Continuous surface charge polarizable continuum models of solvation. I. General formalism. The Journal of Chemical Physics 2010, 132(11), 114110.
26. Kresse, G., Furthmüller, J., Efficient iterative schemes for ab initio total-energy calculations using a plane-wave basis set. Physical Review B 1996, 54(16), 11169-11186.
27. Kresse, G., Furthmüller, J., Efficiency of ab-initio total energy calculations for metals and semiconductors using a plane-wave basis set. Computational Materials Science 1996, 6(1), 15-50.
28. Kresse, G., Hafner, J., Ab initio molecular-dynamics simulation of the liquid-metal--amorphous-semiconductor transition in germanium. Physical Review B 1994, 49(20), 14251-14269.
29. Kresse, G., Hafner, J., Ab initio molecular dynamics for liquid metals. Physical Review B 1993, 47(1), 558-561.
30. Perdew, J.P., Burke, K., Ernzerhof, M., Generalized Gradient Approximation Made Simple. Physical Review Letters 1996, 77(18), 3865-3868.
31. Perdew, J.P., Burke, K., Ernzerhof, M., Generalized Gradient Approximation Made Simple [Phys. Rev. Lett. 77, 3865 (1996)]. Physical Review Letters 1997, 78(7), 1396-1396.
32. Kresse, G., Joubert, D., From ultrasoft pseudopotentials to the projector augmented-wave method. Physical Review B 1999, 59(3), 1758-1775.
33. Blöchl, P.E., Projector augmented-wave method. Physical Review B 1994, 50(24), 17953-17979.

34. Grimme, S., Ehrlich, S., Goerigk, L., Effect of the damping function in dispersion corrected density functional theory. Journal of Computational Chemistry 2011, 32(7), 1456-1465.
35. Methfessel, M., Paxton, A.T., High-precision sampling for Brillouin-zone integration in metals. Physical Review B 1989, 40(6), 3616-3621.
36. Anfuso, C.L., Xiao, D., Ricks, A.M., Negre, C.F.A., Batista, V.S., Lian, T., Orientation of a Series of CO2 Reduction Catalysts on Single Crystal TiO2 Probed by Phase-Sensitive Vibrational Sum Frequency Generation Spectroscopy (PS-VSFG). The Journal of Physical Chemistry C 2012, 116(45), 24107-24114.
37. Ge, A., Rudshteyn, B., Videla, P.E., Miller, C.J., Kubiak, C.P., Batista, V.S., Lian, T., Heterogenized Molecular Catalysts: Vibrational Sum-Frequency Spectroscopic, Electrochemical, and Theoretical Investigations. Accounts of Chemical Research 2019, 52(5), 1289-1300.
38. Vanselous, H., Videla, P.E., Batista, V.S., Petersen, P.B., Distinct Binding of Rhenium Catalysts on Nanostructured and Single-Crystalline TiO2 Surfaces Revealed by Two-Dimensional Sum Frequency Generation Spectroscopy. The Journal of Physical Chemistry C 2018, 122(45), 26018-26031.
39. Adamo, C., Barone, V., Exchange functionals with improved long-range behavior and adiabatic connection methods without adjustable parameters: The mPW and mPW1PW models. The Journal of Chemical Physics 1998, 108(2), 664-675.
40. Ditchfield, R., Hehre, W.J., Pople, J.A., Self-Consistent Molecular-Orbital Methods. IX. An Extended Gaussian-Type Basis for Molecular-Orbital Studies of Organic Molecules. The Journal of Chemical Physics 1971, 54(2), 724-728.
41. Francl, M.M., Pietro, W.J., Hehre, W.J., Binkley, J.S., Gordon, M.S., DeFrees, D.J., Pople, J.A., Self-consistent molecular orbital methods. XXIII. A polarization-type basis set for second-row elements. The Journal of Chemical Physics 1982, 77(7), 3654-3665.
42. Gordon, M.S., The isomers of silacyclopropane. Chemical Physics Letters 1980, 76(1), 163-168.
43. Hariharan, P.C., Pople, J.A., The influence of polarization functions on molecular orbital hydrogenation energies. Theoretica chimica acta 1973, 28(3), 213-222.
44. Hariharan, P.C., Pople, J.A., Accuracy of AH n equilibrium geometries by single determinant molecular orbital theory. Molecular Physics 1974, 27(1), 209-214.
45. Hehre, W.J., Ditchfield, R., Pople, J.A., Self—Consistent Molecular Orbital Methods. XII. Further Extensions of Gaussian—Type Basis Sets for Use in Molecular Orbital Studies of Organic Molecules. The Journal of Chemical Physics 1972, 56(5), 2257-2261.
46. Rassolov, V.A., Pople, J.A., Ratner, M.A., Windus, T.L., 6-31G* basis set for atoms K through Zn. The Journal of Chemical Physics 1998, 109(4), 1223-1229.
47. Rassolov, V.A., Ratner, M.A., Pople, J.A., Redfern, P.C., Curtiss, L.A., 6-31G* basis set for third-row atoms. Journal of Computational Chemistry 2001, 22(9), 976-984.
48. Hay, P.J., Wadt, W.R., Ab initio effective core potentials for molecular calculations. Potentials for the transition metal atoms Sc to Hg. The Journal of Chemical Physics 1985, 82(1), 270-283.
49. Hay, P.J., Wadt, W.R., Ab initio effective core potentials for molecular calculations. Potentials for K to Au including the outermost core orbitals. The Journal of Chemical Physics 1985, 82(1), 299-310.
50. Hay, T.H.D.J.a.P.J., Modern Theoretical Chemistry 1977, 3, 1-27.
51. Wadt, W.R., Hay, P.J., Ab initio effective core potentials for molecular calculations. Potentials for main group elements Na to Bi. The Journal of Chemical Physics 1985, 82(1), 284-298.
